# Epiclomal: Probabilistic clustering of sparse single-cell DNA methylation data

**DOI:** 10.1371/journal.pcbi.1008270

**Published:** 2020-09-23

**Authors:** Camila P. E. de Souza, Mirela Andronescu, Tehmina Masud, Farhia Kabeer, Justina Biele, Emma Laks, Daniel Lai, Patricia Ye, Jazmine Brimhall, Beixi Wang, Edmund Su, Tony Hui, Qi Cao, Marcus Wong, Michelle Moksa, Richard A. Moore, Martin Hirst, Samuel Aparicio, Sohrab P. Shah

**Affiliations:** 1 Department of Statistical and Actuarial Sciences, University of Western Ontario, London, ON, Canada; 2 Department of Molecular Oncology, British Columbia Cancer Research Centre, Vancouver, BC, Canada; 3 Department of Pathology and Laboratory Medicine, University of British Columbia, Vancouver, BC, Canada; 4 Genome Science and Technology Graduate Program, University of British Columbia, Vancouver, BC, Canada; 5 Department of Statistics and Department of Computer Science, University of British Columbia, Vancouver, BC, Canada; 6 Department of Microbiology and Immunology and Michael Smith Laboratories, University of British Columbia, Vancouver, BC, Canada; 7 Genome Sciences Centre, BC Cancer, Vancouver, BC, Canada; 8 Department of Epidemiology and Biostatistics, Memorial Sloan Kettering Cancer Center, New York, NY, USA; Queen’s University, CANADA

## Abstract

We present Epiclomal, a probabilistic clustering method arising from a hierarchical mixture model to simultaneously cluster sparse single-cell DNA methylation data and impute missing values. Using synthetic and published single-cell CpG datasets, we show that Epiclomal outperforms non-probabilistic methods and can handle the inherent missing data characteristic that dominates single-cell CpG genome sequences. Using newly generated single-cell 5mCpG sequencing data, we show that Epiclomal discovers sub-clonal methylation patterns in aneuploid tumour genomes, thus defining epiclones that can match or transcend copy number-determined clonal lineages and opening up an important form of clonal analysis in cancer. Epiclomal is written in R and Python and is available at https://github.com/shahcompbio/Epiclomal.

## Author summary

DNA methylation is an epigenetic mark that occurs when methyl groups are attached to the DNA molecule, thereby playing decisive roles in numerous biological processes. Advances in technology have allowed the generation of high-throughput DNA methylation sequencing data from single cells. One of the goals is to group cells according to their DNA methylation profiles; however, a major challenge arises due to a large amount of missing data per cell. To address this problem, we developed a novel statistical model and framework: Epiclomal. Our approach uses a hierarchical mixture model to borrow statistical strength across cells and neighboring loci to accurately define cell groups (clusters). We compare our approach to different methods on both synthetic and published datasets. We show that Epiclomal is more robust than other approaches, producing more accurate clusters of cells in the majority of experimental scenarios. We also apply Epiclomal to newly generated single-cell DNA methylation data from breast cancer xenografts. Our results show that methylation-based clusters can mirror or in some instances transcend the clusters defined by single-cell copy number analysis. This illustrates the importance of single-cell DNA methylation analysis in understanding cellular heterogeneity in cancer.

## Introduction

DNA methylation of the fifth cytosine position (5mC) is a well studied epigenetic mark that plays decisive roles in the regulation of cell transcriptional programs [1]. In mammals, 5mC occurs mainly at CpG dinucleotides [2] whose distribution is clustered within regions of the genome called CpG islands (CGIs). Bisulfite mediated conversion of 5mC to uracil, referred to as bisulfite sequencing, has been a key tool for quantifying genome-wide DNA methylation at single-cytosine resolution. Advances in technology and laboratory protocols have made it possible to generate high-throughput sequencing data for individual cells [3–6]. In particular, single-cell whole-genome bisulfite sequencing (sc-WGBS) techniques have been developed to assess the epigenetic diversity of a cell population [7, 8]. Because of the limited amount of DNA material, the generated sc-WGBS data are usually sparse, that is, data from many CpG sites are missing and/or are subject to sequencing error. Therefore, there is a great need to develop statistical and computational methods to cluster cells according to their DNA methylation profiles and dealing with the extreme sparsity of the data. The resulting clusters can be used for identification of cancer tumor cell subpopulations [9–11], detection of previously unknown cell types as well as deeper characterization of known ones [12–14], and imputation of missing CpG data by enabling information to be pooled across cells within the same cluster [15].

An increasing amount of sc-WGBS data has been generated from various cell types, including mouse embryonic stem cells [16, 17], human hematopoietic stem cells [7, 12], human hepatocellular carcinomas [11], mouse hepatocytes and fibroblasts [13], human and mouse brain cells [14], and human cell lines [18]. To assess the epigenetic diversity in these different cell populations, a variety of non-probabilistic methods have been considered. Smallwood *et al*. [16] proposed a sliding window approach to compute methylation rates of CpG sites across the genome followed by complete-linkage hierarchical clustering considering Euclidean distances and the most variable sites. Angermueller et al. [17] computed the mean methylation levels across gene bodies and as in [16], clustered the cells using hierarchical clustering and only the most variable genes. Farlik *et al*. [12] clustered cells based on their average methylation over different sets of transcription factor binding sites also using hierarchical clustering. Gravina *et al*. [13] considered the sliding window approach of [16] to compute methylation rates and used principal component analysis to visually assess clusters of cells. Hou *et al*. [11] considered the CpG-based Pearson correlation between pairs of cells followed by hierarchical clustering. Luo *et al*. [14] first applied a hierarchical clustering method called BackSPIN [19] to bin-based methylation rates, followed by cluster merging using gene body methylation levels. Mulqueen *et al*. [18] used NMF (non-negative matrix factorization, [20]) and tSNE [21] for dimensionality reduction, followed by DBSCAN [22] for clustering. Hui *et al*. [7] proposed PDclust, a genome-wide pairwise dissimilarity clustering strategy that leverages the methylation states of individual CpGs. Recently, Kapourani and Sanguinetti [15] proposed a probabilistic clustering method based on a hierarchical mixture of probit regression models and focused their evaluation on missing CpG data imputation. Angermuller *et al*. [23] also proposed a deep learning approach for CpG missing data imputation, but did not address the clustering problem.

Despite the considerable diversity in clustering approaches, there is still a great need for probabilistic, model-based approaches to simultaneously cluster sc-WGBS data while also inferring the missing methylation states. Because such methods enable statistical strength to be borrowed across cells and neighbouring CpGs by assuming that data within the same cell cluster and genomic region share the same model distribution parameters, we surmise that they should provide more robust inference than non-probabilistic methods.

**Fig 1:**
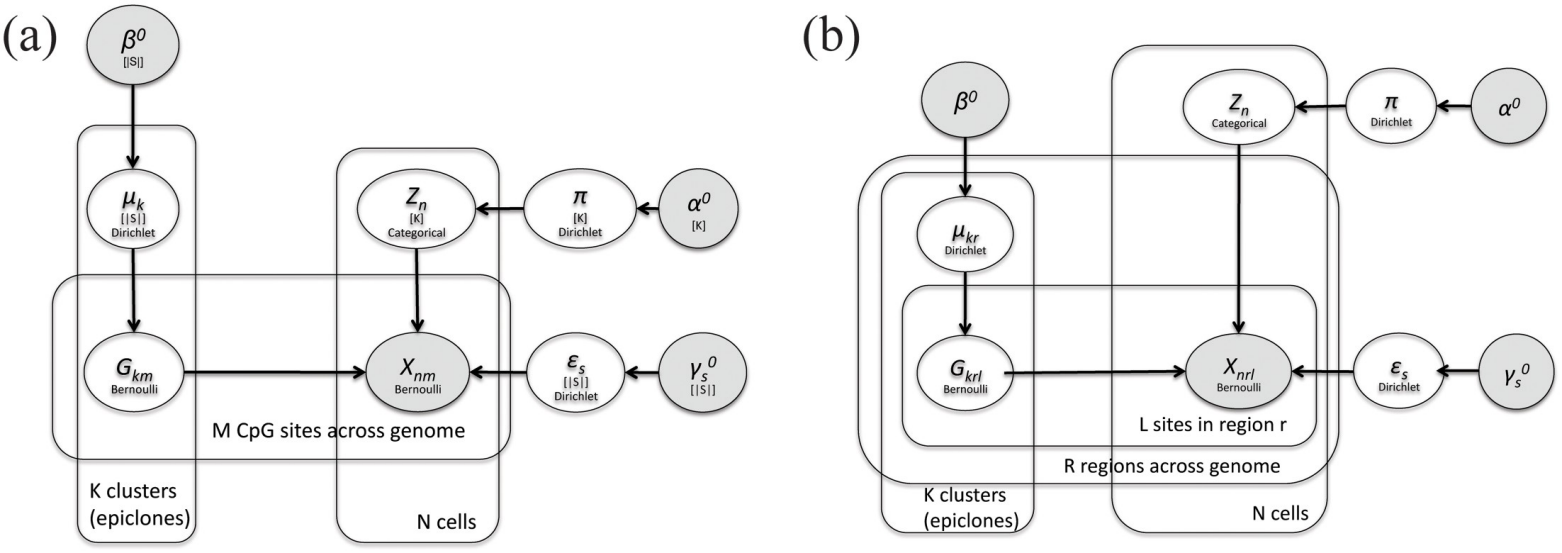
(a) EpiclomalBasic and (b) EpiclomalRegion graphical models. In (a), the shaded node *X*_*nm*_ denotes the observed methylation state at CpG site *m* of cell *n*. In (b), we take into account the region location of each CpG and let the shaded node *X*_*nrl*_ denote the observed methylation state at CpG site *l* of region *r* of cell *n*. Both *X*_*nm*_ and *X*_*nrl*_ take values in S={unmethylated,methylated} or simply S={0,1}. In (a) and (b), the unshaded *Z*_*n*_ node corresponds to the latent variable (with a value in {1, …, *K*}) indicating the true cluster population (epiclone) for cell *n*. The *G*_*km*_ and *G*_*krl*_ unshaded nodes in (a) and (b) respectively are the latent variables with values in S that correspond to the true hidden CpG epigenotypes for each epiclone *k*. The unshaded *μ*, *π*, and *ϵ* nodes in both (a) and (b) correspond to the unknown model parameters, which under the Bayesian paradigm have prior distributions with fixed hyperparameters described by the shaded nodes with the 0 superscript. The distribution assumed for each variable or parameter is written within its node. The edges of the graphs depict dependencies. The plates depict repetitions. In EpiclomalBasic (a), true hidden epigenotypes share the same probability distribution across all CpG sites in the same epiclone (*G*_*km*_ ∼ Bernoulli(*μ*_*k*_)). In EpiclomalRegion (b), true hidden epigenotypes follow a Bernoulli distribution with probability parameters that vary across regions (*G*_*krl*_ ∼ Bernoulli(*μ*_*kr*_)).

**Fig 2:**
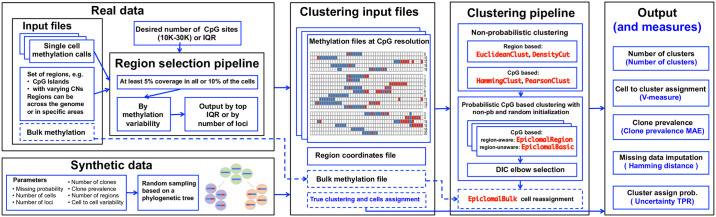
The three components of our proposed framework. *Input data and pre-processing*: data from regions of interest are extracted from methylation call files, which can be filtered to keep only data from regions with a desired amount of missing data and methylation level IQR. A synthetic data pipeline is also provided to simulate data under different parameters. *Clustering*: cells are clustered using different non-probabilistic clustering methods, with results that will then be used as initial values for Epiclomal methods. *Output and performance measures*: different metrics are provided to evaluate the output of each method when true cluster assignments are known.

In this work, we propose Epiclomal, a probabilistic algorithm to cluster sparse CpG-based DNA methylation data from sc-WGBS. Our approach is based on a hierarchical mixture model (see the graphical models in Fig 1), which pools information from observed data across all cells and neighbouring CpGs to infer cell-specific cluster assignments and their corresponding hidden methylation profiles. Epiclomal is part of a novel comprehensive statistical and computational framework (Fig 2) that includes data pre-processing, different clustering methods corresponding to previously proposed approaches [7, 11, 16–18], plotting, and quantitative performance evaluation measures to analyze the results. We use our framework to present an assessment of clustering methods over previously published and synthetic data sets, plus a novel large-scale sc-WGBS data set from breast cancer xenografts [10, 24] generated using state-of-the-art methodology [7].

## Results

### Overview of Epiclomal

Epiclomal is a clustering method based on a hierarchical mixture of Bernoulli distributions. It is given a sparse matrix of *N* rows (cells) and *M* columns (CpG sites), in which each entry is either 0 (unmethylated), 1 (methylated), or missing. The distribution of the observed data *X*_*nm*_ for each CpG site *m* from cell *n* depends on the latent cell-specific cluster assignment *Z*_*n*_ and the corresponding true hidden methylation state (epigenotype) at that CpG, *G*_*km*_ (Fig 1a). We use a Variational Bayes (VB) algorithm (Methods, subsection Model and inference) with random and informed initializations to infer not only cell-to-cluster assignments, but also the true hidden cluster-specific epigenotypes *G*_*k*1_, …, *G*_*kM*_ for each cluster *k*, for *k* = 1, …, *K*. We run Epiclomal considering *K* from 1 to a maximum number of possible clusters and choose the best *K* along with the best clustering assignments as the combination that minimizes the deviance information criterion (DIC, [25]) using an elbow plot selection procedure (Methods, subsection Initialization and choice of *K*).

Epiclomal has two variants: EpiclomalBasic (Fig 1a) and EpiclomalRegion (Fig 1b). While EpiclomalBasic imposes less structure on the model by assuming that the true hidden methylation states share the same distribution across all the CpG sites considered, EpiclomalRegion allows their distribution to vary across genomic functional regions such as CGIs. Although computationally more expensive than EpiclomalBasic, EpiclomalRegion leads to a model that better reflects the expected behaviour of the real data. Bulk data can be used to reassign cells to the EpiclomalRegion clusters using an algorithm that stochastically reassigns cells to clusters while trying to best match the cumulative CpG states of all cells to the corresponding bulk CpG state. This extension is called EpiclomalBulk (Methods, subsection EpiclomalBulk).

Epiclomal is then incorporated into the computational framework presented in Fig 2 and described in what follows.

### Overview of proposed framework

#### Input data and pre-processing

Our framework (Fig 2) can take as input either real or synthetic data. For real data, we take files with CpG methylation calls across the genome from individual cells and extract data from defined regions of interest (e.g., CGIs, gene bodies, and differentially methylated regions). CpGs exhibiting partially methylated calls (median percentage < 1.35 over observed sites for all datasets, Table A in S1 Material) are assigned a value of one (methylated state) if the corresponding methylation fraction was ≥ 0.5 and a value of zero (unmethylated state) otherwise. Because some CpG sites do not exhibit variation and therefore are uninformative for clustering, our framework optionally allows selection of specific regions. One can then consider the data from all regions of interest or apply our region selection pipeline to use data from a subset of those regions. Our proposed selection pipeline first keeps the regions with at least 5% coverage in all or 10% of cells and then selects regions with the most variable methylation levels across cells (using the interquartile range, or IQR), optionally controlling for a desired number of CpG loci. If bulk methylation data are available, our framework can take them as input and use them to inform inference.

For synthetic data, we provide a pipeline that generates single-cell methylation data considering various parameters (e.g., missing proportion, number of cells, and number of loci), assuming that true cluster methylation profiles arise from a phylogenetic process with loci changing methylation states at each new cluster generation (Section 2 in S1 Material). This process is motivated by tumour clonal composition theory [9, 10], in which clonal sub-populations arise from a hierarchical ancestor-descendant phylogenetic process. Note that our proposed synthetic data generator does not simulate data according to our model because Epiclomal is unaware of phylogenetic dependencies.

#### Cluster initialization

Given methylation calls and genomic coordinates of retained regions, we first cluster cells according to various non-probabilistic methods. The results will then be used as initial values for Epiclomal as well as for comparison. We deployed two types of non-probabilistic clustering methods: region- and CpG-based (see Methods, subsection Non-probabilistic clustering methods).

In the region-based approaches, EuclideanClust and DensityCut, we cluster cells considering as input the mean methylation level of each region. EuclideanClust is based on the approaches of [16] and [17] and uses hierarchical clustering with Euclidean distances. DensityCut [26] is a density-based clustering method applied after dimensionality reduction; this resembles the dimensionality reduction technique (NMF [20] + tSNE [21]) followed by a different density-based clustering algorithm (DBSCAN [22]) used by Mulqueen *et al*. [18].

In the CpG-based approaches, Hammingclust and PearsonClust, we consider the methylation state of each individual CpG. HammingClust uses hierarchical clustering with Hamming distances, the same as in PDclust [7]. PearsonClust applies hierarchical clustering using Pearson correlation values, which is equivalent to the approach used in [11].

To find the optimal number of clusters, DensityCut includes its own automatic method, whereas for the hierarchical clustering methods we use the Calinski-Harabasz (CH) index [27]. Our pipeline runs Epiclomal using the results of the non-probabilistic methods as initial values along with a set of random initial values and chooses the best configuration, as explained in the “Overview of Epiclomal” section.

#### Output and performance measures

For all clustering methods, our framework outputs predictions of cell-to-cluster assignments, number of clusters, and cluster (or epiclone) distribution frequencies (i.e., the proportion of cells assigned to each cluster). In addition, for Epiclomal, we obtain the estimated missing CpG values and the cell-to-cluster assignment posterior probabilities.

When ground-truth clustering is available, we also output a performance evaluation measure for each of the five predictions described above (Fig 2 and Section 3 in S1 Material). The V-measure [28] evaluates the cell-to-cluster assignments and is a score between zero and one, where one stands for perfect clustering and zero for random cell-to-cluster assignments. The V-measure captures the homogeneity and completeness of a clustering result. To satisfy the homogeneity criterion, a clustering procedure must assign only those cells that are members of a single group to a single cluster. Completeness is satisfied if all those cells that are members of a single group are assigned to a single cluster. The harmonic mean of homogeneity (*h*) and completeness (*c*) gives rise to the V-measure ($V=\frac{2hc}{h+c}$), and even a small percentage of misclassified cells can significantly affect it.

We also report the predicted number of clusters and the mean absolute error (MAE) between true and predicted cluster frequencies. In addition, when applying Epiclomal on synthetic data, we consider the Hamming distance as the proportion of discordant entries between true and inferred vectors of methylation states. We also compute for Epiclomal the uncertainty true positive rate of cluster assignment probabilities, that is, how well the uncertainty is estimated for cells whose membership is unclear due to missing data.

### Epiclomal outperforms other methods on synthetic data

To evaluate the performance of our proposed methods over a wide range of characteristics, we generated a large number of synthetic datasets and applied our Epiclomal approaches (EpiclomalRegion, EpiclomalBasic, and EpiclomalBulk), as well as the four non-probabilistic methods (EuclideanClust, DensityCut, HammingClust, and PearsonClust) to each generated data set.

We considered several experiments, where in each one we varied one of eight parameters while keeping the others fixed, as indicated in Table 1. For each setting, we generated 30 input datasets and ran Epiclomal with a total of 300 informed and random VB initializations. Then we computed the V-measure along with the other quantities included in our framework to assess method performance.

**Table 1:** Varying parameters and their ranges for synthetic data simulation. For each experiment, we varied one parameter and kept the others fixed. Note that varying the number of regions is equivalent to varying region size because the total number of loci is fixed. Unless otherwise specified, the fixed parameters are: missing proportion 0.8, region size 100, number of cells 100, proportion of cell-to-cell variability 0, number of epiclones 3, equal epiclone frequencies (1/3), number of loci 10,000, and number of regions different between clusters 1. For the cell-to-cell variability experiment (Fig 4c), we used 25 regions to have a larger number of loci that differed between clusters. For the number of epiclones experiment (Fig 4d) and the epiclone frequency experiment (Fig 4e), we used 500 cells to allow for enough cells to be represented in each case. For the number of loci experiment (Fig 4f), we also varied the number of regions to keep the differences among clusters fixed (e.g., 50 regions for 5,000 loci, 5,000 regions for 500,000 loci).

Varying parameter	Varying range
Missing proportion	0.5 to 0.95
Number of regions	25 to 200
Number of cells	12 to 2500
Cell-to-cell variability	0 to 0.3
Number of clusters (epiclones)	1 to 10
Epiclone frequencies	balanced to very unbalanced
Number of loci	5 000 to 500 000
Number of regions different between clusters	1 to 6

**Fig 3:**
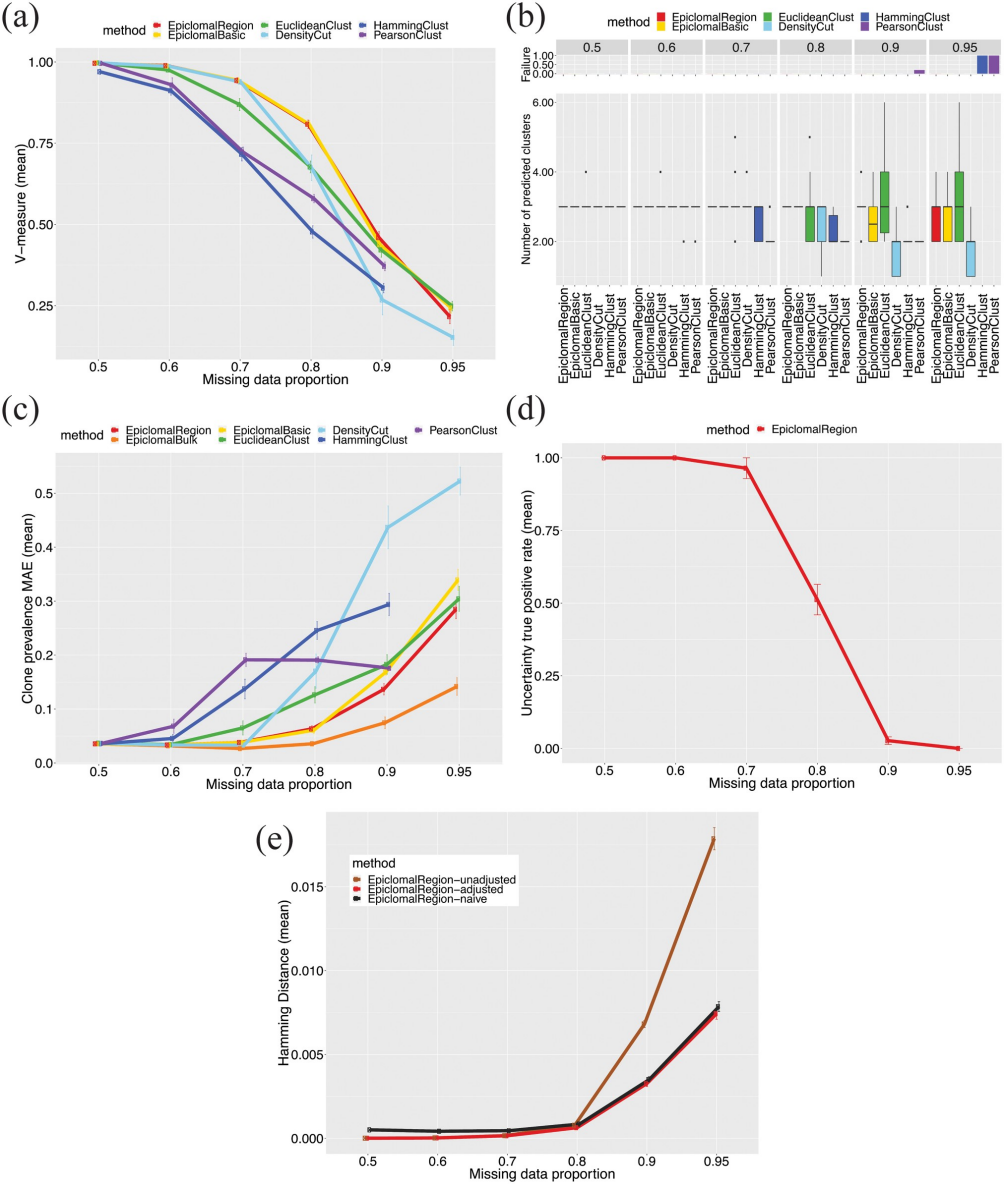
Simulation results when varying the missing data proportion. We report mean results produced by Epiclomal and the non-probabilistic methods taken over 30 randomly generated synthetic datasets: (a) V-measure; (b) Number of predicted clusters (true is 3); the top panel shows the proportion of data sets for which a method failed to produce a result; (c) Epiclone frequency (prevalence) MAE (mean absolute error); (d) Uncertainty true positive rate; and (e) Hamming distance for three variants of EpiclomalRegion inferred methylation states: unadjusted, adjusted, and naive (see Sections 1.2 and 3.4 in S1 Material). The vertical bars correspond to one standard deviation above and below the mean value.

Fig 3 shows the results when the proportion of missing data is varied from 0.5 to 0.95. Our proposed probabilistic Epiclomal methods give better or comparable V-measures (panel a) with overall more correct values for the number of clusters (*K* = 3, panel b) than the non-probabilistic methods, which tend to overestimate (EuclideanClust) or underestimate (PearsonClust, HammingClust, and DensityCut) the number of clusters. PearsonClust and HammingClust fail to produce results in the case of 0.95 proportion of missing data. Using bulk data via EpiclomalBulk shows improvement in estimating cluster frequencies, especially when the missing data proportion is large (0.9 and 0.95, panel c). The cluster assignment uncertainty is well estimated by EpiclomalRegion for up to 0.7 missing proportion; however, it drops rapidly for 0.8 and 0.9 missing proportion (panel d).

**Fig 4:**
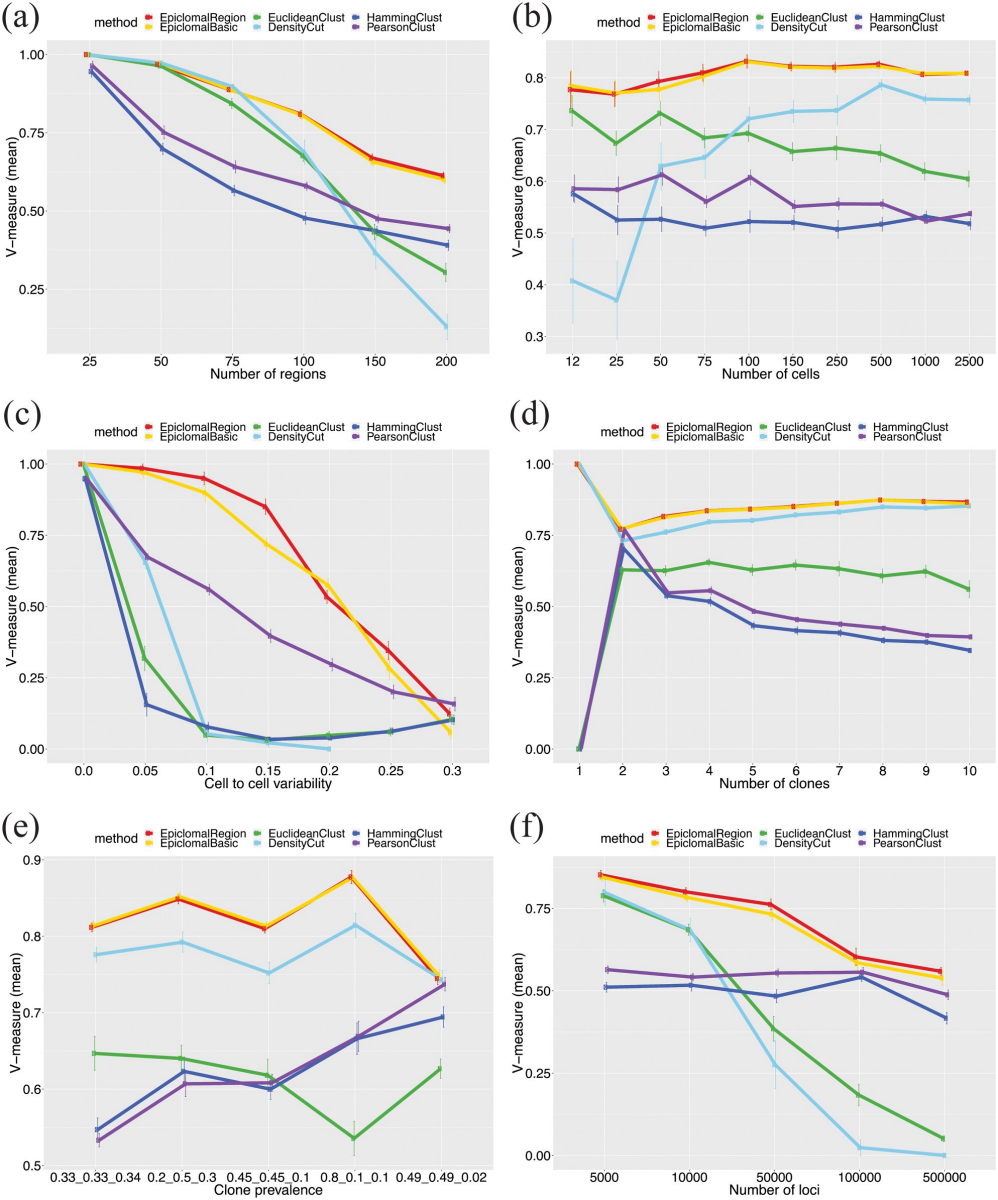
Predicted cell-to-cluster assignments on synthetic data. We report mean V-measures produced by Epiclomal and the non-probabilistic methods taken over 30 randomly generated synthetic data sets, when we vary by: (a) the number of regions, (b) the number of cells, (c) the cell-to-cell variability, (d) the number of clones, (e) the cluster frequencies (prevalences), and (f) the number of loci. The vertical bars correspond to one standard deviation above and below the mean value. The Epiclomal methods outperformed the other methods in all cases.

Fig 4 shows that Epiclomal results in a better V-measure than the non-probabilistic methods in all the remaining experimental scenarios with a fixed missing proportion of 0.8 (see also Figures C to I in S1 Figs). All methods perform worse when the problem is more difficult, such as when decreasing the number of different loci among clusters (Fig 4a and Figure I in S1 Figs) or increasing cell-to-cell variability (Fig 4c). Increasing the number of cells (Fig 4b) does not improve the V-measure, except for DensityCut, but it does reduce its variability. The Epiclomal methods are more robust to an increasing number of epiclones (Fig 4d) and a change in epiclone frequencies (Fig 4e). When increasing the number of loci (Fig 4f), the performance of HammingClust and PearsonClust remains somewhat constant, but the other methods show a decreasing pattern of performance. However, the Epiclomal methods still perform better for all numbers of loci considered than all the other methods. Therefore, this provides support to a strategy for selecting a smaller number of loci (under 50,000) in order to keep the true signal and eliminate noise when analyzing a real data set.

**Fig 5:**
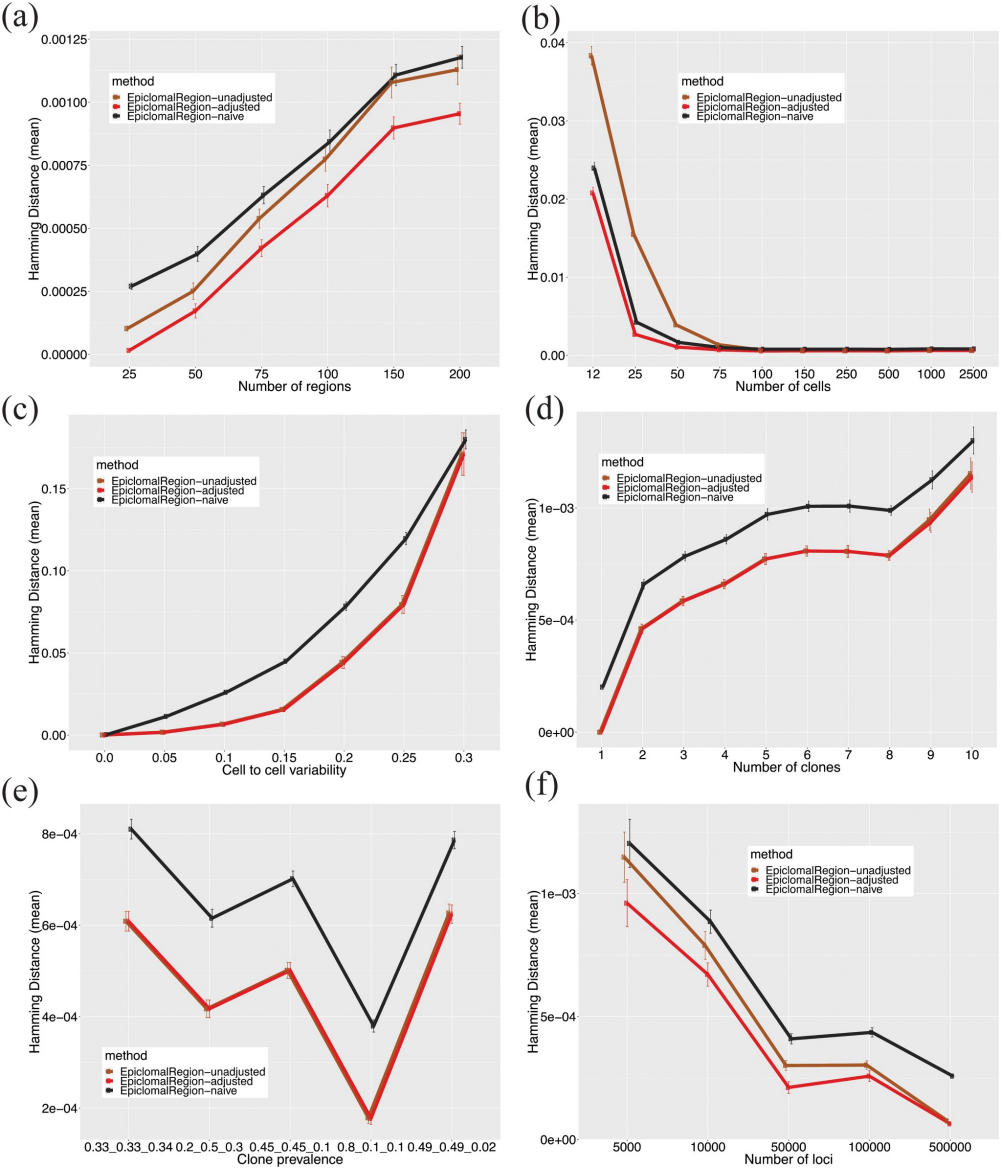
Imputation results on synthetic data. Average hamming distance for three variants of EpiclomalRegion inferred methylation states: unadjusted, adjusted, and naive (see Sections 1.2 and 3.4 in S1 Material) when varying: (a) the number of regions, (b) the number of cells, (c) the cell-to-cell variability, (d) the number of clones, (e) the cluster frequencies (prevalences), and (f) the number of loci. The vertical bars correspond to one standard deviation above and below the mean value.

Figs 3e and 5 reveal that Epiclomal can generally impute CpG methylation states more correctly than a naive imputation (see Section 1.2 in S1 Material) for the same clustering result.

### Epiclomal recapitulates methylation subgroups from public datasets

We further assessed the performance of our methods on three published sc-WGBS datasets [11, 12, 16] and compared our results with the clustering results reported in each paper. Experimental validation of epiclones is often difficult, and therefore when working with cells from different known types or treatment conditions, authors expect their clusters to somewhat reflect the epigenetic diversity of those types [12, 16]. In [11], there were no predefined cell sub-populations; however the authors considered gene expression and copy number changes to further support their findings.

Table 2 shows that these datasets display a variety of characteristics, with missing data proportions varying from 0.54 to 0.98. Table A in S1 Material presents the results of analysing the three datasets for non-binary methylation states. We observed extremely small percentages of CpGs with partially methylated states for all datasets analyzed, with a median < 1.35% across cells when using all observed CpG sites for all datasets. When using only CpG sites with at least two reads aligned to them, we observed a median < 2.25% for all datasets except for Farlik2016 (the sparsest dataset), which had a slightly larger median of 5.13%.

**Table 2:** Summary of the real data sets used in this work. Column descriptions (in order of appearance) are as follows: (1) data set names corresponding to three published data sets and the new in-house data set; (2) type of cells in each data set; (3) number of cells considered for each data set, which varied from tens to hundreds of cells; (4) number of clusters, as reported in the respective published papers, NA (not available) for our in-house data set; (5) genomic functional regions considered for each data set, which were the same as in the original papers when applicable, CGI stands for CpG Islands, TFBS stands for Transcription Factor Binding Sites; (6) missing data proportion for each data set for the 10,000 loci filtered input and varying from 0.69 to 0.89; (7) number of loci for the largest input data sets obtained by including all regions with methylation IQR ≥ 0.01; these varied from one-quarter million to 1 million CpG sites; (8) missing data proportion for the largest input data sets, which varied from 0.54 to 0.98.

Data set	Cell type	# cells	# clusters	Regions	Miss 10K	Nloci IQR ≥ .01	Miss IQR ≥ .01
Smallwood2014 [16]	mouse embryonic stem cells	32	2	CGI	0.69	786 620	0.54
Hou2016 [11]	human hepatocellular carcinomas	25	2	CGI	0.87	255 136	0.90
Farlik2016 [12]	human hematopoietic cells	122	6	TFBS	0.89	512 153	0.98
InHouse	human xenografted cancer cells (3 patients)	558	NA	CGI	0.82	1 019 956	0.79

**Fig 6:**
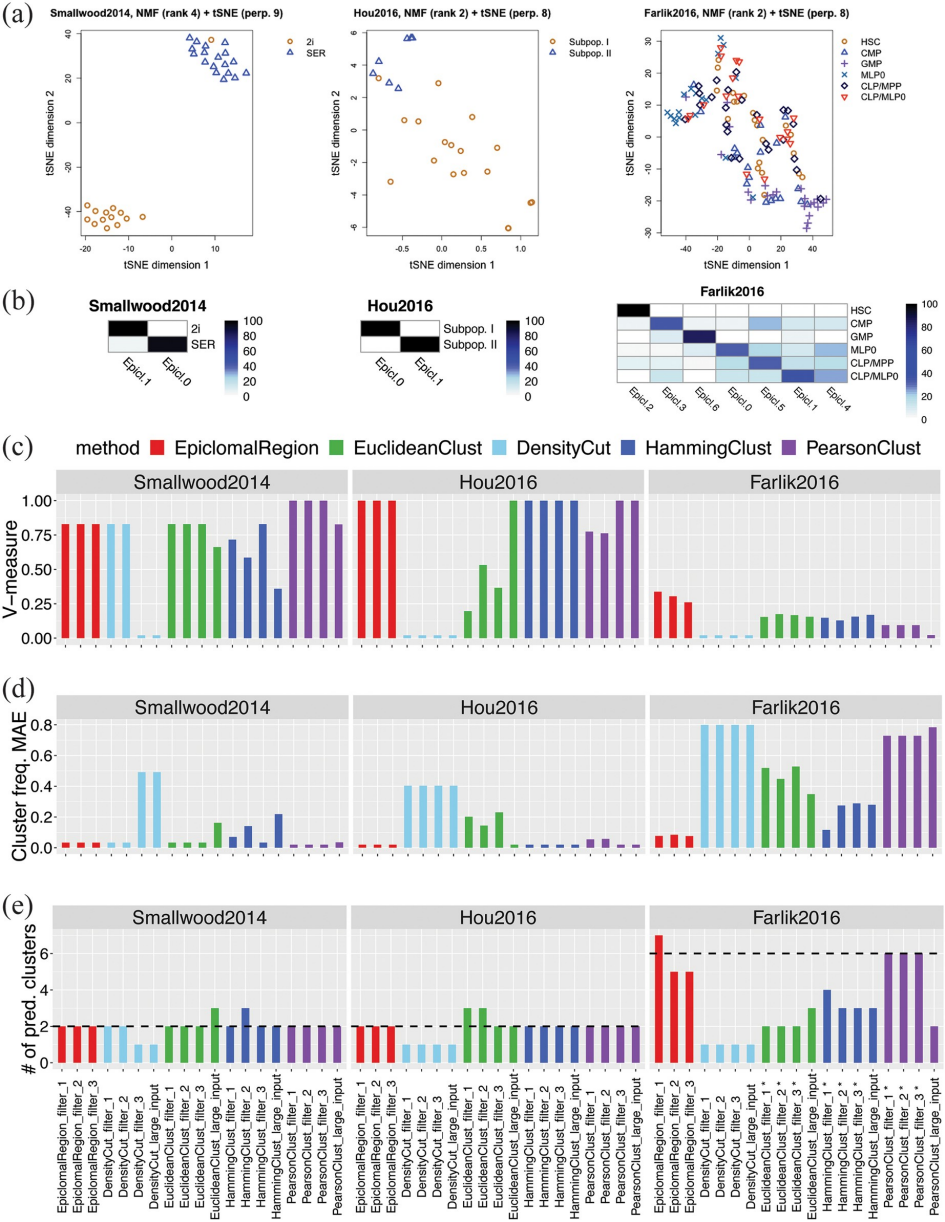
Results on the real data sets. (a) Dimensionality reduction visualization plots showing the clustering reported in the published papers on the ≈ 10,000 loci processed data sets. (b) Co-clustering between the real data published clusters on the rows and EpiclomalRegion predictions on the columns. Each entry *a*_*ij*_ is the percentage of cells in published class *i* that are present in predicted cluster *j*, with the rows summing up to 100%. A perfect agreement would result in a square matrix with a black diagonal. (c) V-measures comparing the cell assignments with the published assignments, with higher values meaning better agreement. (d) Cluster frequencies mean absolute error, comparing the inferred proportions of clusters with the published proportions, with lower values meaning better agreement. (e) Number of predicted clusters. The horizontal dashed lines correspond to the published number of clusters; bars closer to this line represent better agreement.

Fig 6a shows a dimensionality reduction visualization using NMF [20] followed by tSNE [21]. Note, however, that this does not clearly separate the clusters, particularly for more challenging data sets, such as Farlik2016. UMAP [29] or simple tSNE did not show better separation; instead, heatmaps of average methylation rates in each cell and genomic region clearly show the specific features of each epiclone (Figures K, L and M in S1 Figs). Fig 6b shows co-clustering plots that summarise the EpiclomalRegion’s cell-to-cluster assignments.

Fig 6c to 6e present the results for cell-to-cluster assignments, cluster frequencies and number of clusters, respectively, of applying our framework to these published datasets considering three filtered inputs with 10,000, 15,000 and 20,000 CpG loci (see Methods for details on pre-processing real data). In addition, we evaluated the usefulness of selecting regions by IQR of mean methylation levels by running all non-probabilistic methods on a large input that filtered out only regions with methylation IQR < 0.01 and also plotted the results in Fig 6c to 6e.

The Smallwood2014 dataset [16] is made up of 32 mouse embryonic stem cells, where 20 cells were cultured in a regular serum medium and 12 cells in a 2i medium inducing hypomethylation. Fig 6 shows good agreement between the clusters inferred by EpiclomalRegion and the ones obtained by [16], with only one discordant cell (V-measure 0.82 for 10,000 loci, Fig 6c and Figure K in S1 Figs). PearsonClust correctly clustered all cells for the three filtered input datasets (V-measure = 1, Fig 6c), but the other non-probabilistic methods misclassified one or two cells.

The Hou2016 dataset [11] contains 25 cells from a human hepatocellular carcinoma tissue sample. We compared our results with the two subpopulations identified by [11] based not only on DNA methylation, but also on copy number and gene expression data. For all input datasets, EpiclomalRegion correctly assigned all cells to their corresponding subpopulations (V-measure = 1, Fig 6).

The Farlik2016 data set [12] contains different types of human hematopoietic cells, totalling 122 cells. We compared our results with the six clusters found by Farlik et al. [12], made up of hematopoietic stem cells (HSC) and progenitor cell types (myeloid, multipotent, and lymphoid progenitor cells). For the input with 10,000 loci, EpiclomalRegion resulted in a V-measure of 0.34, with seven predicted clusters (Fig 6c to 6e). As stated before, the V-measure can be significantly affected by a small percentage of misclassified cells. Therefore, even though the V-measure is low, Fig 6b shows good agreement between Epiclomal clustering and the clustering reported by Farlik *et al*.

Fig 6c to 6e show that EpiclomalRegion generally outperformed the non-probabilistic methods on V-measure, cluster frequency mean absolute error, and number of correctly predicted clusters. In addition, because Epiclomal is based on a Bayesian inference approach, posterior means and standard deviations of model parameters can be obtained as illustrated in Figure J in S1 Figs, which presents EpiclomalRegion inferred posterior mean methylation probabilities along with standard deviations across regions and clusters (i.e., the posterior means and standard deviations of *μ*_*kr*_ for all *k* and *r*; see Fig 1b and Eq. (14) in S1 Material) for the filtered input of about 10,000 loci.

### Epiclomal reveals copy number-dependent and copy number-independent epiclones in breast cancer

**Fig 7:**
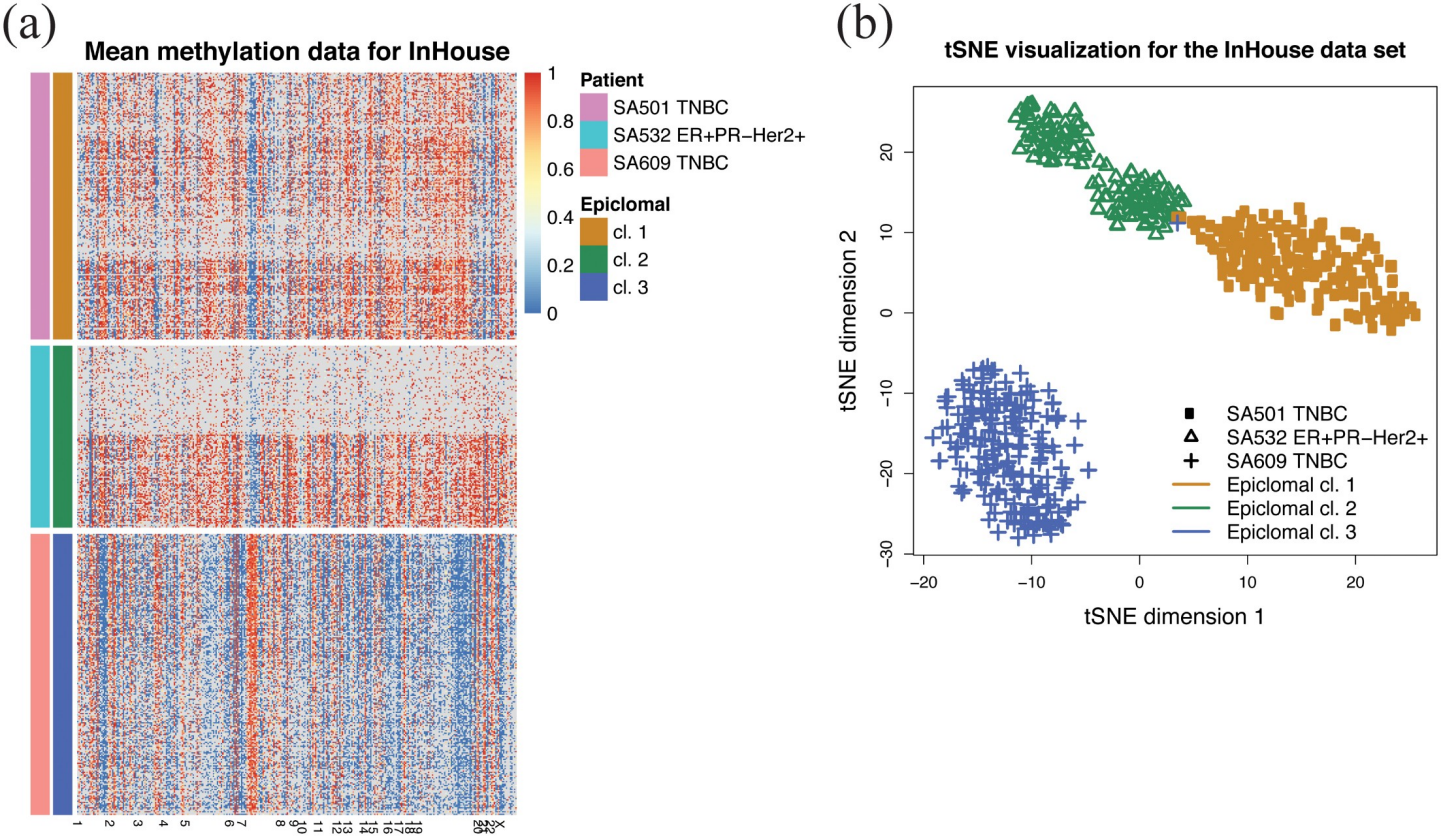
Visualization of the InHouse clusters. (a) EpiclomalRegion clustering, with data filtered to include the most variable CGIs and obtain ≈ 15,000 loci (327 CGIs, cell average missing proportion 0.82, 558 cells). EpiclomalRegion obtained 3 clusters. Rows are cells, and columns are CGIs. (b) tSNE dimensionality reduction and color-coding of the Epiclomal clusters onto the tSNE 2-dimensional space.

Having verified the performance of Epiclomal on synthetic data and public domain datasets, we set out to perform epiclone group discovery on single-cell epigenomes generated in-house on a range of patient-derived breast tumour xenografts. First, to illustrate the scalability of Epiclomal with aneuploid single-cell cancer epigenomes, we analysed 558 tumour xenograft single epigenomes (called InHouse data) from two patients (SA501 and SA609) with triple-negative breast cancer and one patient (SA532) with ER+PR-Her2+ breast cancer (Table B in S1 Material) sequenced using the PBAL method [7]. Fig 7a and 7b show the heatmap and t-SNE visualization respectively for the InHouse data along with the clustering results of EpiclomalRegion, which resulted in three clusters, one for each patient. Fig 7a shows the methylation differences between the three patients, with SA609 having a highly different methylation profile than the other two. Note that two different experimental plates of markedly different missing proportions for SA532 (Fig 7a) resulted in visually distinct subclusters in Fig 7b, potentially affecting density-based approaches. Indeed, DensityCut clustered these plates into two different clusters, yet Epiclomal was robust to this batch effect.

We next focused our analysis on one of the three patient-derived xenografts mentioned above, which was previously characterized with whole-genome sequencing (WGS) [10] and single-cell WGS [4] (patient SA501 in Table B in S1 Material). Breast cancers often exhibit whole chromosome gains and losses (in addition to sub-chromosomal aneuploidy), especially of the X chromosome, which provides a strong methylation signal. As previously described, this patient-derived xenograft (PDX) underwent copy number clonal dynamics between passages, with clones losing one copy of X eventually dominating the populations of later passages. Patient tumour cells at diagnosis were mouse xenografted and serially transplanted over generations. Then sc-WGBS data from passages 2, 7, and 10 were generated using the PBAL protocol [7]. After filtering out cells that did not pass quality control upon alignment (see Methods), we obtained a final sc-WGBS dataset of 244 single cells over three passages. We considered as initial regions the set of differentially methylated CGIs found when comparing bulk BS-seq data from passages 1 and 10 (see Methods). We then applied non-negative matrix factorization (NMF [18, 20, 30]) to the region mean methylation data of all 244 cells as a feature selection strategy obtaining a final input set of 94 regions (see S1 Table for their coordinates). Over all 94 regions, chromosome X contained the most differentially methylated regions of any single chromosome (29 out of 94; Figures N and O in S1 Figs).

**Fig 8:**
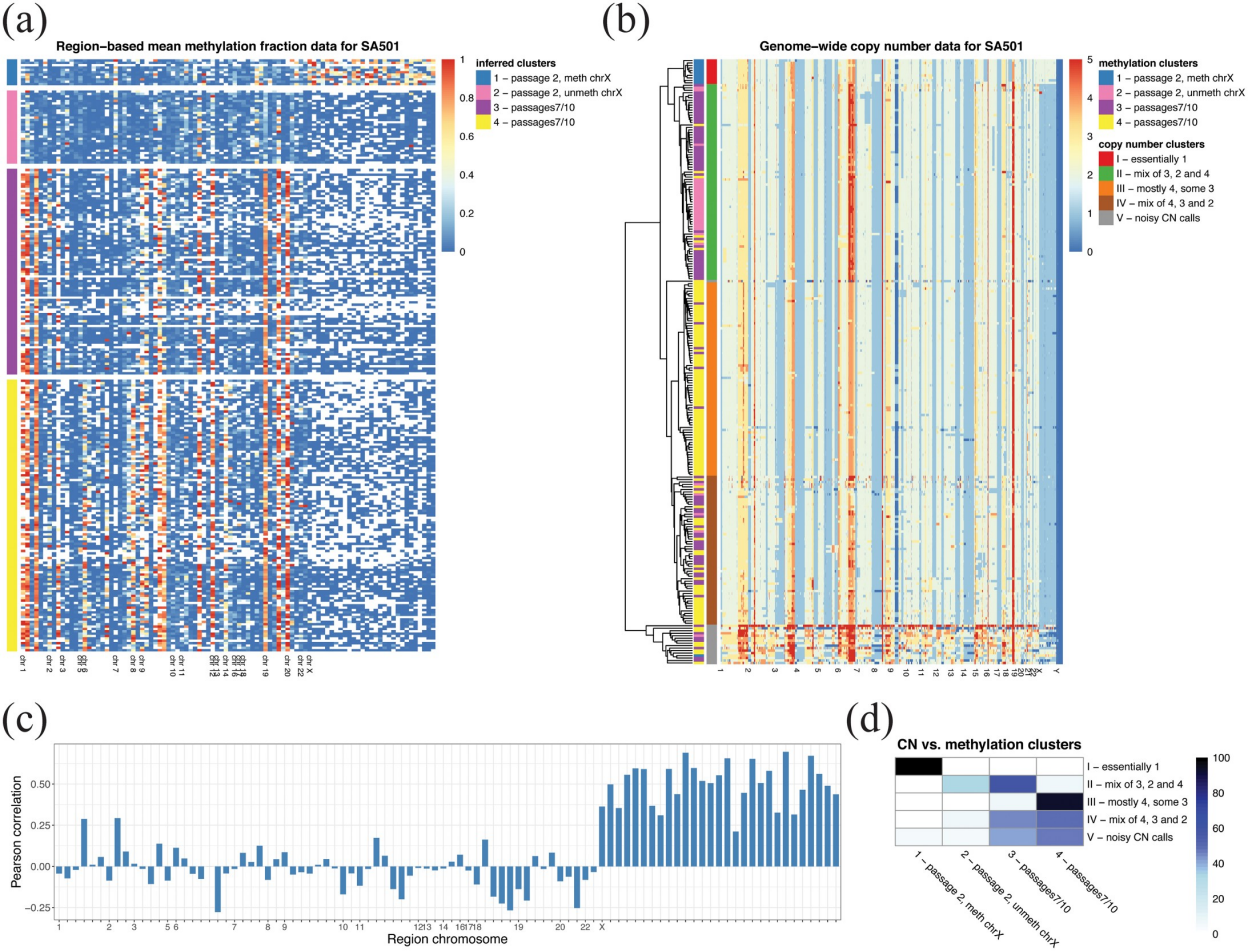
Results for patient SA501. (a) Mean methylation level for each of the 94 NMF-selected regions (CGIs) for patient SA501 across all cells ordered according to the four methylation clusters found using EpiclomalRegion. Rows are cells, and columns are CGIs. (b) Inferred genome-wide copy numbers for the same cells as in (a) clustered using a ward.D2 hierarchical clustering method and Euclidean copy number distances. Note that copy number 5 means five or more copies. To call copy number changes, we used the methylation sc-WGBS data. Only one epiclone and one copy number clone matched, the remaining clones transcended each other. (c) Pearson correlation between mean methylation data and copy number data in each of the 94 regions. There was correlation in chromosome X, but not in the autosomal chromosomes. (d) Heatmap showing the percentage of cells in the copy number clusters (rows) that are in the methylation clusters (columns); rows sum up to 100.

Using these 94 regions, EpiclomalRegion clustered the cells (Fig 8a) into four epiclones: two primarily containing passage 2 cells, and two containing a mix of passage 7 and 10 cells (EpiclomalBasic produced the same results). The distribution of posterior cluster assignment probabilities (*p*) indicates that most cells were classified with *p*>0.9, except for two cells that were assigned to Cluster 3 with probabilities of 0.73 and 0.69.

Inspection shows that Cluster 1 contains 10/40 passage 2 cells (and 1 passage 7 cell) with unmethylated features, except for chromosome X regions, which are mainly methylated. This is consistent with a small population of passage 2 cells presenting two copies of the X chromosome (Fig 8b) and normal X-inactivation mechanism for which the X-inactive copy exhibits most CGIs hypermethylated [31]. Cluster 2 contains the remaining 30/40 passage 2 cells (and one passage 10 cell), but with unmethylated chromosome X regions, which is compatible with the loss of one copy of X (see also Fig 8b) indicating that either the active X was the inherited copy or that the inactive X was demethylated. At later passages, several autosomal regions became methylated (see, for example, chromosomes 1, 9, 12, 19, and 20 in S2 Table). In addition, we identified three main regions that are methylated only in some of the later passage cells (see also S2 Table), resulting in two different epiclones, each containing a mix of passage 7 and 10 cells (cluster 3 containing 53/98 passage 7 cells and 34/106 passage 10 cells; and cluster 4 containing 44/98 passage 7 cells and 71/106 passage 10 cells).

These observations suggest that some chromosomal regions, such as X, may show strong copy number influence on CpG states, whereas others may differ in CpG state, but be unrelated to copy number state in aneuploid genomes. Therefore, we next investigated possible correlations between methylation and copy number alterations derived from the same sc-WGBS data (see Methods). A systematic comparison shows that indeed the average methylation levels and copy number states across cells for each of the 94 regions (Fig 8b) were only highly correlated (Pearson correlation >0.5; Fig 8c) for the X chromosome. This implies that epiclones may transcend copy number-defined clones in the studied SA501 PDX.

Indeed, when we compared the four epiclones with the four sc-PBAL copy number (CN) clones, we noticed that they can match or transcend each other as follows (Fig 8b and 8d): epiclone 1 with methylated regions in the X chromosome matches exactly CN clone I having two copies of the X chromosome, which shows a strong relationship between the presence of the second copy of the X chromosome and the methylation pattern. However, 26/31 passage-2 cells with all 94 regions unmethylated from epiclone 2 are found in CN clone II, which also contains 47/87 cells from epiclone 3 and 6/115 cells from epiclone 4, even though these have several regions that are methylated. Finally, epiclone 3 transcends CN clones II (47/87 cells from epiclone 3 are in CN clone II) and IV (27/87 cells from epiclone 3 are in CN clone IV), and epiclone 4 transcends CN clones III (72/115 cells from epiclone 4 are in clone III) and IV (29/115 cells from epiclone 4 are in CN clone IV). Taken together, these data show for the first time with single-cell methylation analysis that epigenetically defined clones may present a different lineage from that of copy number-defined clonal architectures, opening up this form of analysis for cancer genomes.

## Discussion

Single-cell CpG genome analysis is currently held back by a dearth of principled methods for handling the features of single-cell methylation data. To this end, we have developed Epiclomal, a probabilistic CpG-based clustering method for clustering sparse sc-WGBS data and elucidating the epigenetic diversity of different types of cell populations. Epiclomal uses a principled Variational Bayes inference method that is robust to the initial starting point, with the optimal clustering being obtained multiple times across independent runs well before our 1000 iteration cut-off (Figure P in S1 Figs). Our method has produced overall better results than non-probabilistic based methods when tested on synthetic data from eight extensive simulation scenarios (Figs 3 and 4, and Figures B to I in S1 Figs) and three comprehensive real data sets (Fig 6). Epiclomal is robust and consistent to subsampling by CpG coverage and number of cells and can generally impute missing CpG methylation values more correctly than a naive imputation for the same clustering result (Figs 3e and 5 and Figure Q in S1 Figs). Importantly, Epiclomal is reliable when the amount of data missing is large and/or varies across cells and can find the true clusters and epiclone frequencies when the signal is subtle, which are both limiting features of current sc-WGBS data.

It is well understood that 5mC distribution in the genome is regionally clustered and that this has implications for computational methods. EpiclomalRegion considers CpG-based methylation dependencies in functional regions and models errors while simultaneously assigning cells to clusters and imputing missing data. It can also use bulk DNA methylation data to improve epiclone frequencies, which are important quantities, particularly for the study of cancer tumour composition. Epiclomal works at the CpG level and hence considers the contribution of every sequenced CpG site in the selected regions, without loss of information by region averaging. Epiclomal not only runs an uninformed clustering method, but also uses the clustering results of four other methods (with more easily added) and a robust model selection strategy to return the best prediction.

Epiclomal is part of an extensive statistical and computational framework that provides interpretable results and five performance measures. It also enables the easy inclusion of novel components in the computational pipeline. Our framework includes a pre-processing step where specific regions can be selected to increase signal and eliminate noise in the input data. Epiclomal obtained better or equal results on the smaller filtered input datasets than on the larger ones, supporting the notion that filtering out the most invariant regions may improve the signal for clustering. In addition, our synthetic experiments as well as the SA501 intra-patient analysis on a well-designed set of differentially methylated regions showed that pre-processing the initial whole-genome data set in a way that keeps the clone differences and eliminates noise is likely to produce better results overall. Our selection strategy has the limitation of possibly removing regions that vary only in a small percentage of cells, which may result in clusters being condensed together. Future work includes a region selection strategy that can increase the signal-to-noise ratio. One approach, for example, would be to consider the variation across sites within regions, so that regions with the same variation pattern across cells could be represented only once in the model by appropriate weights in the data log-likelihood function.

Although epigenomic states are of importance in cancer biology, to date very few single-cell whole-genome bisulfite datasets have been generated on aneuploid cancer genomes. In this study, Epiclomal was used with a large (598 genomes, Table B in S1 Material) new sc-WGBS data set generated by the PBAL method to demonstrate how epiclones and copy number-determined clones differ. Epiclomal was able to identify known and novel CpG methylation substructures that could not be identified by non-probabilistic distance-based methods due to the missing data inherent in sc-WGBS. Specifically, the separation between the two passage 7/10 subclusters was not found by any of the non-probabilistic methods we considered, even when a larger set of regions was used. This demonstrates that sophisticated modeling of missing data and appropriate region selection are necessary to clearly separate possibly weak biological signals.

The ability to identify CpG-defined sub-clones, or epiclones, made it possible for the first time to compare a copy number-determined lineage with an epigenetically defined lineage. It is expected that for certain regions of the genome, for example where allelic hemi-methylation occurs, changes in chromosomal copy number would strongly pattern 5mC CpG status. Indeed, we observed this with subclones of a breast cancer PDX (SA501), where biallelic X chromosome clones that were present in early passages contained epiclones with and without CpG methylation. In contrast, we observed in the PDX studied that clones defined by autosomal copy number aberrations can exhibit quite distinct epiclone structures, leading to the notion that in some cases, epiclone-defined lineage will transcend that of copy number-defined lineage. This is an expected result since DNA methylation mediates transcriptional cellular memory and therefore cell states that are not “clonal” in the sense of arising from cell division associated mutational processes. This has important implications for the study of cancer evolution and clonal states because a failure to include epigenetic states will under-represent the cellular population structures of interest. Further work is required to define the scope and nature of epiclone-defined versus copy number clone-defined cellular lineages in cancer.

## Methods

### Proposed probabilistic approach—Epiclomal

#### Model and inference

Our proposed methodology extends the approach of [32] to single-cell DNA methylation data. In what follows, we describe our model and the Bayesian inference technique for the case we call EpiclomalRegion, which is based on the assumption that the probability of a given locus being methylated depends on the genomic region where that locus is situated and that loci in the same genomic region share the same methylation probability. The EpiclomalBasic approach is a special case of EpiclomalRegion that is obtained by assuming that all loci belong to one single region sharing the same probability of being methylated and therefore can be obtained by setting *R* = 1 in all derivations below. See the graphical models in Fig 1.

Let us consider a set of *R* regions in the genome (e.g., CGIs, gene bodies). Let *X*_*nrl*_ be the observed methylation status (or epigenotype) for cell *n* at locus *l* of region *r*, for *n* = 1, …, *N*, *r* = 1, …, *R*, and *l* = 1, …, *L*_*r*_. Our approach allows the set of loci with observed data to vary across cells, but for simplicity, we write our model and inference derivations assuming that there are data for all loci in all cells, i.e., assuming complete data. Each *X*_*nrl*_ takes a value in S={unmethylated,methylated} or simply S={0,1}.

Let $X_{nr}={\left(X_{nr1},\ldots ,X_{nrL_{r}}\right)}^{T}$ be the vector of observed data for region *r* in cell *n*, and let $X_{n}={\left(X_{nr}^{T},\ldots ,X_{nR}^{T}\right)}^{T}$ be the vector of all observed data for cell *n*. Assume that $X_{nr1},\ldots ,X_{nrL_{r}}$ are independent for all *n* and *r*. Suppose that there are *K* ≪ *N* vectors of true hidden methylation states shared across the cells. Let *Z*_*n*_ with values in {1, …, *K*} be the hidden variable indicating the true cluster (epiclonal) population of cell *n*. It is assumed that *Z*_1_, …, *Z*_*N*_ are independent with *P*(*Z*_*n*_ = *k*) = *π*_*k*_ such that $\sum_{k=1}^{K}\pi_{k}=1$. If *Z*_*n*_ = *k*, then the distribution of **X**_*n*_ depends on the *k*-th vector of true hidden epigenotypes $G_{k}={\left(G_{k1}^{T},\ldots ,G_{kR}^{T}\right)}^{T}$, where $G_{kr}={\left(G_{kr1},\ldots ,G_{krL_{r}}\right)}^{T}$. We assume that $G_{kr1},\ldots ,G_{krL_{r}}$ are independent for all *k* and *r*, with *P*(*G*_*krl*_ = *s*) = *μ*_*krs*_ such that $\sum_{s\in S}\mu_{krs}=1$, that is, *G*_*krl*_ follows a categorical (Bernoulli) distribution with parameter set $\mu_{kr}=\left\{\mu_{krs}:s\in S\right\}$. Therefore, given the true cluster assignment and the corresponding true hidden methylation states, the observed data **X**_*nr*_ are independent, with *X*_*nrl*_ following a categorical distribution with parameters depending on the hidden true state at locus *l* of region *r* for cluster population *k*, that is,

$$\begin{array}{c} P\left(X_{nrl}=t|Z_{n}=k,G_{krl}=s\right)=\epsilon_{st}\;\text{with}\;\sum_{t\in S}\epsilon_{st}=1. \end{array}$$ (1)

We can also interpret the probability in (1) as a misclassification error, which in this context is related to sequencing error.

Let Θ be the set containing all the model parameters, i.e., Θ = {***μ***, ***ϵ***, ***π***}, where

- $\mu ={\left(\mu_{1}^{T},\ldots ,\mu_{K}^{T}\right)}^{T}$ with $\mu_{k}={\left(\mu_{k1}^{T},\ldots ,\mu_{kR}^{T}\right)}^{T}$ and $\mu_{kr}=\left\{\mu_{krs}:s\in S\right\}$;
- $\epsilon =\{\epsilon_{s}:s\in S\}$ with $\epsilon_{s}=\left\{\epsilon_{st}:t\in S\right\}$ and
- ***π*** = (*π*_1_, …, *π_K_*)^*T*^.

To infer Θ and the hidden states **Z** = (*Z*_1_, …, *Z*_*n*_)^*T*^ and **G** = {**G**_1_, …, **G**_*K*_}, we adopt a Bayesian approach and derive a Variational Bayes (VB) algorithm [33] to approximate the posterior distribution of Θ, **Z**, and **G** given the observed data **X** = {**X**_1_, …, **X**_*N*_}, *P*(**Z**, **G**, Θ|**X**) by finding the Variational Distribution (VD), *q*(**Z**, **G**, Θ) with the smallest Kullback-Leibler divergence to the posterior *P*(**Z**, **G**, Θ|**X**), which is equivalent to maximizing the evidence lower bound (ELBO) given by

$$\begin{array}{c} \text{ELBO}\left(q\right)=E[\log P(X,Z,G,\Theta )]-E[\log q(Z,G,\Theta )]. \end{array}$$ (2)

See [34] for more details. We assume the following prior distributions for the parameters in Θ.

- $p\left(\mu \right)=\prod_{k=1}^{K}p\left(\mu_{k}\right)=\prod_{k=1}^{K}\prod_{r=1}^{R}p\left(\mu_{kr}\right)$, where ***μ****_kr_* ∼ Dirichlet(*β*^0^)
- $p\left(\epsilon \right)=\prod_{s\in S}p\left(\epsilon_{s}\right)$, where $\epsilon_{s}∼\text{Dirichlet}\left(\gamma_{s}^{0}\right)$
- ***π*** ∼ Dirichlet(*α*^0^)

Please refer to Section 1.1 in S1 Material for all steps of the proposed VB algorithm for inferring **Z**, **G**, and Θ.

#### Initialization and choice of *K*

Because maximizing the ELBO, as given in (2), is generally a non-convex optimization problem [34], it can lead to a local optimum. To avoid this problem, it is crucial to initialize the proposed VB algorithm properly. Therefore, we developed the following initialization framework to tackle this challenge. We ran the Variational Bayes algorithm a maximum number of times *T* (we used *T* = 1000 for the real data sets and *T* = 300 for the synthetic data sets). We started from different initial posterior cluster assignment probabilities, $\pi_{n}^{*}$, for each cell *n* (for the other two posterior parameters that needed to be initialized, we used their corresponding prior hyperparameters, that is, $\gamma_{s}^{*(0)}=\gamma_{s}^{0}$ and $\beta_{kr}^{*(0)}=\beta^{0}$; see Section 1.1 in S1 Material). In other words, each vector $\pi_{n}^{*}$ of length *K* will have *K* − 1 values of 0 and one value of 1, corresponding to the initial cluster assignment for that cell. Most initializations are uniformly random, but informed starting values often lead to better results. Therefore, for all analyses, we used the following initialization strategy. First, we ran EuclideanClust and if the hierarchical clustering was successful, we cut the hierarchical tree at 1, 2…*K* clusters, obtaining the first *K* initial points. Then we did the same for HammingClust and PearsonClust, obtaining 2 × *K* more initial points. Finally, we added the prediction made by DensityCut. Note that initializations from more clustering methods can be easily added to our framework.

In our analyses, we used *K* = 10 for all synthetic and real data sets. Therefore, a maximum of *I* = 31 initializations came from the non-probabilistic methods. The remaining *T* − *I* VB runs were initialized randomly, with each initial number of clusters being a number chosen uniformly at random between 1 and *K*. For each run, the VB algorithm returned a number of recommended clusters *c* ≤ *K* and the corresponding cell-to-cluster assignments. With this strategy, we obtained a more uniform number of clusters across all runs than if we had used the same *K* for each run. Therefore, our strategy resembles a BIC or AIC selection criterion in which we would perform a roughly equal number of runs for each possible number of recommended clusters.

After obtaining the *T* runs (this was done in parallel on a computing cluster), we have for each run the number of recommended clusters *c* ≤ *K* and the computed DIC score that takes into account the likelihood of the model as well as the model complexity [25]. Then, for each *c*, we compute the minimum DIC obtained for all runs that recommended *c* clusters, and we plot the DIC curve, as in Figure A in S1 Figs.

Now, with a DIC curve, the elbow point can be found as follows. We draw a line from the first to the last point of the curve and then find the DIC point that is the farthest away from that line. Sometimes, the DIC curve is not a smooth decreasing function, but instead it can increase and decrease. Therefore, we decided to consider only the part of the curve with DIC values decreasing by at least a small percentage threshold (0.2%), which is the green line in Figure A in S1 Figs. We then find the elbow for this part of the curve, which corresponds to the best choice of the number of clusters and it is shown by the red line in Figure A in S1 Figs. The DIC-elbow selection strategy can be used as an automatic way to select the best run. However, visual inspection of the DIC-elbow can sometimes help choose the best thresholds.

#### EpiclomalBulk

Often, bulk CpG-level methylation data are produced, that is, a vector of natural numbers, representing the number of methylated cytosines for each CpG, from 0 to the read depth *D* (e.g., *D* = 60). For instance, a value of 0 means that we expect no cell to be methylated (all are unmethylated) at that CpG site. A value of 60 means that we expect all the cells to be methylated, and a value of 30 means that roughly half the cells are methylated and half are unmethylated. Therefore, given the cell-to-cluster assignments and the corresponding imputed methylation values, we can compute a score that tells us how well the given imputed values match the bulk data (for each CpG site, we just have to count the number of cells that are methylated and then divide by the number of cells and multiply by *D*).

With this bulk-based score function, we designed a stochastic local search algorithm that starts from a given configuration (which is EpiclomalRegion’s best result), keeps the number of clusters fixed, and randomly reassigns “uncertain cells” to one of their “candidate clusters”. The “uncertain cells” and the “candidate clusters” are obtained as described in Section 3.5 in S1 Material. Only the CpGs in the regions that make the clusters different are considered. If the new score is better than before, we always keep it; if it is not, we keep it only 20% of the time to help the algorithm escape local minima. We repeat this strategy for 10 iterations and return the combination of new cell-to-cluster assignments and imputed methylation states that gives the best score.

### Non-probabilistic clustering methods

#### EuclideanClust

EuclideanClust is a region-based method in which we first compute for each cell the mean methylation level of each region of interest. Because of the sparsity of the data, we cluster the cells, taking as input data not the original matrix of mean methylation levels, but instead we apply complete-linkage hierarchical clustering to the symmetric matrix of Euclidean distances between every pair of cells with a dissimilarity matrix based also on Euclidean distances. EuclideanClust is similar to the approach used by Smallwood *et al*. [16] and Angermuller *et al*. [17], with the difference that the regions are defined differently in our case (functional genomic regions) versus Smallwood (sliding windows across the genome) and Angermuller *et al*. (gene bodies). We use the Calinski-Harabasz (CH) index [27] to automatically choose the number of clusters that best fits the data.

#### DensityCut

As in EuclideanClust, we first compute for each cell the mean methylation level of each region of interest. We then use principal component analysis as a dimensionality reduction technique with a maximum of 20 first principal components and apply DensityCut, a density-based clustering algorithm proposed by [26], to the resulting principal component scores.

#### HammingClust

This method is a CpG-based method because it considers data from all individual CpGs from all regions of interest to cluster the cells. Because of the sparsity of the data, similarly to EuclideanClust, clustering is done by first calculating Hamming distance-based dissimilarities (proportion of discordant positions) between each pair of cells and then applying Ward’s linkage hierarchical clustering with Euclidean distances on the matrix of Hamming-based dissimilarities. PDclust as proposed by Hui *et al*. [7] produces the same dendrogram as HammingClust because it consists of the same steps and dissimilarities, except that PDclust uses percentages of discordant positions and HammingClust proportions. PDClust does not include an automatic method to select the optimal number of clusters, but Hammingclust uses the CH index for that purpose. In addition, HammingClust has the advantage of being implemented in C++ within R, resulting in much faster computation than PDclust, which is implemented solely in R.

#### PearsonClust

PearsonClust is also a CpG-based approach much like HammingClust, except that instead of Hamming and Euclidean distances, it is based entirely on Pearson correlation. In other words, it first computes the Pearson correlation between every pair of cells and then applies Ward’s linkage hierarchical clustering with again a Pearson-based dissimilarity matrix on the initial correlation matrix. This method is equivalent to the approach used by Hou *et al*. [11] with the addition of the CH index to select the best clustering partition.

#### Pre-imputation of missing values for non-probabilistic methods

Sometimes the input matrix to the non-probabilistic methods is too sparse, and either hierarchical clustering or the CH-index method for choosing the number of clusters will fail to produce results. For the synthetic data, we simply report this as a failure in order to understand what characteristics of the input data set make this failure happen. However, for real data sets, we try to run the hierarchical methods without pre-imputation, and if they fail, we rerun them after pre-imputation; see the star-labelled runs in Fig 6.

### Pre-processing of real data

We pre-processed real data sets using the first part of our proposed framework. For each data set, we started by considering all regions of the corresponding type presented in the fifth column of Table 2. Then, after eliminating the empty regions across all cells, we also removed regions with an average missing proportion across all cells greater than or equal to 95%. Next, we kept the most variable regions (as measured by IQR of mean methylation levels) that would produce three filtered inputs with 10,000, 15,000 and 20,000 loci respectively.

### In-house sc-WGBS data generation

#### Biospecimen collection and ethical approval

Tumour fragments from women diagnosed with breast lump undergoing surgery or diagnostic core biopsy were collected with informed consent according to procedures approved by the Ethics Committees at the University of British Columbia. Patients in British Columbia were recruited and samples collected under the tumor tissue repository (TTR-H06-00289) protocol that falls under the UBC BCCA Research Ethics Board.

#### Tissue processing

The tumor materials were processed as described in [10]. Briefly, the tumor fragments were minced finely with scalpels and then mechanically disaggregated for one minute using a Stomacher 80 Biomaster (Seward Limited, Worthing, UK) in 1-2 mL cold DMEM-F12 medium. Aliquots from the resulting suspension of cells and clumps were used for xenotransplants.

#### Xenografting

Xenograft samples were transplanted and passaged as described in [10]. Female immune compromised, NOD/SCID interleukin-2 receptor gamma null (NSG) and NOD Rag-1 null interleukin-2 receptor gamma null (NRG) mice were bred and housed at the Animal Resource Centre (ARC) at the British Columbia Cancer Research Centre (BCCRC) supervised by the Aparicio lab. Surgery was carried out on mice between the ages of 8-12 weeks. The animal care committee and animal welfare and ethical review committee of the University of British Columbia (UBC) approved all experimental procedures. For subcutaneous transplants, mechanically disaggregated cells and clumps of cells were resuspended in 100-200*μ*l of a 1:1 v/v mixture of cold DMEM/F12: Matrigel (BD Biosciences, San Jose, CA, USA). Eight- to twelve-week-old mice were anesthetised with isoflurane, after which the cell/clumps suspension was injected under the skin on the flank using a 1 ml syringe and a 21-gauge needle.

#### Histopathological review

On histopathological review, two out of three, i.e., SA501 and SA609, patient-derived xenografts used in this study were triple negative breast cancers (TNBC). On immunohistochemistry, they were found to be receptor negative breast cancer subtype. SA532 was a ER+PR-HER2+ xenograft. A pathologist reviewed the slides.

#### Cell preparation and dispensing

Xenograft tissues were dissociated to cells as described in [4] before dispensing single cells into the wells of 384 well plates using a contactless piezoelectric dispenser (sciFLEXArrayer S3, Scienion) with real-time cell detection in the glass capillary nozzle (CellenOne).

#### sc-WGBS experimental protocol

The Post-Bisulfite Adapter Ligation (PBAL) protocol described in [7] was used to obtain in-house sc-WGBS data.

#### Data alignment and methylation calls

One lane of paired end sequencing was used to create each single cell library. Trim Galore (v0.4.1) and Cutadapt(v1.10) were used for quality and adapter trimming. Libraries were aligned to a GRCh37-lite reference using Novoalign (v3.02.10) in bisulfite mode and converted to BAM format and sorted using Sambamba (v0.6.0). Bam files were annotated for duplicates using Picard Tools’ MarkDuplicates Jar (v1.92). Novomethyl (v1.10) was used in conjunction with in-house scripts (samtools v1.6 and bedtools v2.25.0) to determine methylation of each CpG as described in Section “NovoMethyl—Analysing Methylation Status” Section of the Novoalign documentation (http://www.novocraft.com/documentation/novoalign-2/novoalign-user-guide/bisulphite-treated-reads/novomethyl-analyzing-methylation-status/).

#### Quality control

Using an in-house script, libraries were filtered according to a delta CT and 100K read count threshold to account for suitable library depth. Libraries over the expected number of copy number variants were filtered out to control for chromothripsis and shattered cells.

#### Copy number calling

Copy number changes for SA501 were called using the same sc-WGBS DNA methylation data (copy number calling from the DLP protocol [4] largely matches the sc-WGBS copy number calling for passage 2). Control Free-c (v7.0) was used to copy number variant call on processed BAMs. The following settings were used: ploidy: 2, window and telocentromeric: 500000, sex: XY, minExpectGC: 0.39 and maxExpectedGC: 0.51.

### In-house bulk whole-genome bisulfite sequencing (SA501, passages 1 and 10)

#### Whole-genome bisulfite library construction for Illumina sequencing

To track the efficiency of bisulfite conversion, 10 ng lambda DNA (Promega) was spiked into 1 *μ*g genomic DNA quantified using Qubit fluorometry and arrayed in a 96-well microtitre plate. DNA was sheared to a target size of 300 bp using Covaris sonication and the fragments end-repaired using DNA ligase and dNTPs at 30 C for 30 min. Repaired DNA was purified using a 2:1 AMPure XP beads-to-sample ratio and eluted in 40 *μ*L elution buffer in preparation for A-tailing; adenosine was then added to the 3’ end of DNA fragments using Klenow fragment and dATP incubated at 37 C for 30 min. Following reaction clean-up with magnetic beads, cytosine methylated paired-end adapters (5’-A^*m*^CA^*m*^CT^*m*^CTTT^*m*^C^*m*^C^*m*^CTA^*m*^CA^*m*^CGA^*m*^CG^*m*^CT^*m*^CTT^*m*^C^*m*^CGAT^*m*^CT-3’ and 3’-GAG^*m*^C^*m*^CGTAAGGA^*m*^CGA^*m*^CTTGG^*m*^CGAGAAGG^*m*^CTAG-5’) were ligated to the DNA at 30°C, 20 min, and adapter-flanked DNA fragments bead purified. Bisulfite conversion of the methylated adapter-ligated DNA fragments was achieved using the EZ Methylation-Gold kit (Zymo Research) following the manufacturer’s protocol. Seven cycles of PCR using HiFi polymerase (Kapa Biosystems) were used to enrich the bisulfite-converted DNA and introduce fault-tolerant hexamer barcode sequences. Post-PCR purification and size-selection of bisulfite-converted DNA were performed using 1:1 AMPure XP beads. To determine final library concentrations, fragment sizes were assessed using a high-sensitivity DNA assay (Agilent) and DNA quantified by Qubit fluorometry. Where necessary, libraries were diluted in elution buffer supplemented with 0.1% Tween-20 to achieve a concentration of 8 nM for Illumina HiSeq2500 flow cell cluster generation.

#### Data alignment and methylation calls

FASTQ files were trimmed with TrimGalore (0.4.1) and then input into Bismark (0.14.4), aligning with bowtie2 (2.2.6). With the output BAM, we used samtools (1.3) to sort by name, fix mates, sort by position, remove duplicates, and then finally sort by name once again and filter out reads with a mapping quality of 10 or less. We then ran the resulting BAM files through the bismark_methylation_extractor script that accompanies Bismark to call methylation sites. All tools were run on all default settings, with changes made only to increase run speed.

#### Differentially methylated CpG Islands

Differentially methylated CpG islands between bulk samples from tumour xenograft passages 1 and 10 were obtained via Fisher’s exact test considering all CpG islands with coverage greater than or equal to than five reads. The Benjamini-Hochberg procedure was used to correct for multiple testing.

## Supporting information

S1 Material Supporting text and tables. (PDF)

S1 Figs Supporting figures. (PDF)

S1 Table Supporting table containing the coordinates of the 94 regions presented in Fig 8. (XLSX)

S2 Table Supporting table containing additional information regarding the regions presented in Fig 8. (XLSX)
