# Supplementary material for: Epiclomal: Probabilistic clustering of sparse single-cell DNA methylation data
Source: PLoS Comput Biol. 2020 Sep 23;16(9):e1008270. doi: 10.1371/journal.pcbi.1008270 (PMC7546467; doi:10.1371/journal.pcbi.1008270)
Supplement: S1 Material — (PDF) [file pcbi.1008270.s001.pdf]

Epiclomal: probabilistic clustering of sparse single-cell DNA methylation data

Supplementary Material

Contents

1 Proposed probabilistic method - Epiclomal **2**

1.1 Variational Bayes (VB) algorithm . . . 2

1.2 Adjustment of imputed CpG states and naive imputation . . . 6

1.3 Computational complexity . . . 7

1.4 Implementation . . . 7

2 Synthetic data generator **7**

3 Evaluation measures **8**

3.1 V-measure . . . 8

3.2 Number of clusters . . . 9

3.3 Cluster frequency mean absolute error . . . 9

3.4 Hamming distance . . . 9

3.5 Uncertainty true positive rate . . . 10

4 Supplementary Tables **11**

1 Proposed probabilistic method - Epiclomal

1.1 Variational Bayes (VB) algorithm

In what follows we describe the main steps of the proposed VB algorithm for inferring $\mathbf{Z}, \mathbf{G}$ and Θ (see Methods, subsection Model and inference in the main text).

Step 1. Variational distribution factorization

We assume the following factorization of the variational distribution (VD):

$$\begin{aligned} q(\mathbf{Z}, \mathbf{G}, \Theta) &\equiv q(\mathbf{Z})q(\mathbf{G})q(\boldsymbol{\mu})q(\boldsymbol{\epsilon})q(\boldsymbol{\pi}) \\ &= \Big[\prod_{n=1}^{N} q(Z_n)\Big]\Big[\prod_{k=1}^{K}\prod_{r=1}^{R}\prod_{l=1}^{L_r} q(G_{krl})\Big]\Big[\prod_{s\in\mathcal{S}} q(\boldsymbol{\epsilon}_s)\Big]\Big[\prod_{k=1}^{K}\prod_{r=1}^{R} q(\boldsymbol{\mu}_{kr})\Big]q(\boldsymbol{\pi}). \end{aligned} \tag{1}$$

Step 2. Derivation of the joint distribution of observed data, hidden variables and parameters

Considering the assumptions previously made (see Methods in the main text) for the observed data, hidden variables and model parameters, we can write the logarithm of the joint distribution of $\mathbf{X}$, $\mathbf{Z}$, $\mathbf{G}$ and the parameters in Θ as

$$\log P(\mathbf{X}, \mathbf{Z}, \mathbf{G}, \Theta) = \log P(\mathbf{X}|\mathbf{Z}, \mathbf{G}, \Theta) + \log P(\mathbf{G}|\Theta) + \log P(\mathbf{Z}|\Theta) + \log P(\Theta), \tag{2}$$

where

$$\log P(\mathbf{X}|\mathbf{Z}, \mathbf{G}, \Theta) = \sum_{n=1}^{N}\sum_{r=1}^{R}\sum_{l=1}^{L_r}\sum_{k=1}^{K}\sum_{s\in\mathcal{S}}\sum_{t\in\mathrm{S}} \mathrm{I}(Z_n = k)\mathrm{I}(X_{nrl} = t)\mathrm{I}(G_{krl} = s)\log\epsilon_{st}; \tag{3}$$

$$\log P(\mathbf{G}|\Theta) = \sum_{k=1}^{K}\sum_{r=1}^{R}\sum_{l=1}^{L_r}\sum_{s\in\mathcal{S}} \mathrm{I}(G_{krl} = s)\log\mu_{krs};$$

$$\log P(\mathbf{Z}|\Theta) = \sum_{n=1}^{N}\sum_{k=1}^{K} \mathrm{I}(Z_n = k)\log\pi_k \text{ and} \tag{4}$$

$$\log P(\Theta) = \log P(\boldsymbol{\epsilon}) + \log P(\boldsymbol{\mu}) + \log P(\boldsymbol{\pi})$$

with

$$\log P(\boldsymbol{\epsilon}) = \sum_{s\in\mathcal{S}}\Big[\sum_{t\in\mathcal{S}}(\gamma_{st}^0 - 1)\log\epsilon_{st} - \log B(\boldsymbol{\gamma}_s^0)\Big]; \tag{5}$$

$$\log P(\boldsymbol{\mu}) = \sum_{k=1}^{K}\sum_{r=1}^{R}\Big[\sum_{s\in\mathcal{S}}(\beta_s^0 - 1)\log\mu_{krs} - \log B(\boldsymbol{\beta}^0)\Big] \text{ and} \tag{6}$$

$$\log P(\boldsymbol{\pi}) \;=\; \sum_{k=1}^{K}(\alpha_k^0 - 1)\log \pi_k - \log B(\boldsymbol{\alpha}^0). \tag{7}$$

The function B in (5)-(7) is the multivariate Beta function, which can be expressed in terms of gamma functions. So, for example,

$$B(\boldsymbol{\alpha}^0) = \frac{\prod_{k=1}^{K}\Gamma(\alpha_k^0)}{\Gamma(\sum_{k=1}^{K}\alpha_k^0)}.$$

Step 3. VD derivation

We now approximate each term in the factorization (1) by calculating the expectation of $\log P(\mathbf{X}, \mathbf{Z}, \mathbf{G}, \Theta)$ over the distribution of all terms except the one of interest. So, for example, we obtain the approximation $q^*(\boldsymbol{\pi})$ for $q(\boldsymbol{\pi})$ by calculating

$$\log q^*(\boldsymbol{\pi}) = \mathbb{E}_{-\boldsymbol{\pi}}\left(\log P(\mathbf{X}, \mathbf{Z}, \mathbf{G}, \Theta)\right) + C,$$

where $-\boldsymbol{\pi}$ indicates that the expectation is taken with respect to the VD of all other terms than $\boldsymbol{\pi}$, i.e., $\mathbf{Z}$, $\mathbf{G}$, $\boldsymbol{\epsilon}$ and $\boldsymbol{\mu}$. See [1] and [2] for more details. Below we present the form of q^* for each term in Eq. (1).

- Approximating $q(\boldsymbol{\pi})$ by $q^*(\boldsymbol{\pi})$

  We find $q^*(\boldsymbol{\pi})$ that satisfies

  $$\log q^*(\boldsymbol{\pi}) = \mathbb{E}_{\mathbf{Z},\mathbf{G},\boldsymbol{\epsilon},\boldsymbol{\mu}}\left(\log P(\mathbf{X}, \mathbf{Z}, \mathbf{G}, \Theta)\right) + C.$$

  As only the terms (4) and (7) in (2) depend on $\boldsymbol{\pi}$ we calculate:

  $$\begin{aligned}\log q^*(\boldsymbol{\pi}) \;&=\; \mathbb{E}_{\mathbf{Z},\mathbf{G},\boldsymbol{\epsilon},\boldsymbol{\mu}}\left(\log P(\mathbf{Z}|\Theta)\right) \;+\; \mathbb{E}_{\mathbf{Z},\mathbf{G},\boldsymbol{\epsilon},\boldsymbol{\mu}}\left(\log P(\boldsymbol{\pi})\right) \;+\; C^* \\ &=\; \sum_{n=1}^{N}\sum_{k=1}^{K}\mathbb{E}_{q^*(Z_n)}\left(\mathrm{I}(Z_n = k)\right)\log \pi_k + \log P(\boldsymbol{\pi}) + C^* \\ &=\; \sum_{k=1}^{K}\log \pi_k\Big[\sum_{n=1}^{N}\mathbb{E}_{q^*(Z_n)}\left(\mathrm{I}(Z_n = k)\right)\Big] + \sum_{k=1}^{K}\log \pi_k(\alpha_k^0 - 1) + C^{**} \\ &=\; \sum_{k=1}^{K}\log \pi_k\Big[\Big(\sum_{n=1}^{N}\mathbb{E}_{q^*(Z_n)}\left(\mathrm{I}(Z_n = k)\right) + \alpha_k^0\Big) - 1\Big] + C^{**}.\end{aligned}$$

  Therefore, $q^*(\boldsymbol{\pi})$ is a Dirichlet distribution with parameters $\alpha^* = (\alpha_1^*, \ldots, \alpha_K^*)^T$, where

  $$\alpha_k^* = \alpha_k^0 + \sum_{n=1}^{N}\mathbb{E}_{q^*(Z_n)}\left(\mathrm{I}(Z_n = k)\right). \tag{8}$$

- Approximating $q(Z_n)$ by $q^*(Z_n)$

  To find $q^*(Z_n)$ we calculate

$$\log q^*(Z_n) = \mathbb{E}_{Z_{i:i\neq n},\mathbf{G},\boldsymbol{\epsilon},\boldsymbol{\mu}}\big(\log P(\mathbf{X},\mathbf{Z},\mathbf{G},\Theta)\big) + C. \tag{9}$$

As only (3) and (4) in (2) depend on Z_n, calculating the approximation in (9) is equivalent to calculating the following:

$$\log q^*(Z_n) = \mathbb{E}_{Z_{i:i\neq n},\mathbf{G},\boldsymbol{\epsilon},\boldsymbol{\mu}}\big(\log P(\mathbf{X}|\mathbf{Z},\mathbf{G},\Theta)\big) + \mathbb{E}_{Z_{i:i\neq n},\mathbf{G},\boldsymbol{\epsilon},\boldsymbol{\mu}}\big(\log P(\mathbf{Z}|\Theta).\big) \tag{10}$$

Note that we can split $\log P(\mathbf{X}|\mathbf{Z},\mathbf{G},\Theta)$ in (3) into two terms: one that depends on Z_n and one that is constant on Z_n, that is,

$$\begin{aligned}\log P(\mathbf{X}|\mathbf{Z},\mathbf{G},\Theta) &= \sum_{r=1}^{R}\sum_{l=1}^{L_r}\sum_{k=1}^{K}\sum_{s\in\mathcal{S}}\sum_{t\in\mathrm{S}} \mathrm{I}(Z_n=k)\mathrm{I}(X_{nrl}=t)\mathrm{I}(G_{krl}=s)\log\epsilon_{st}\\ &+ \sum_{i\neq n}\sum_{r=1}^{R}\sum_{l=1}^{L_r}\sum_{k=1}^{K}\sum_{s\in\mathcal{S}}\sum_{t\in\mathrm{S}} \mathrm{I}(Z_i=k)\mathrm{I}(X_{irl}=t)\mathrm{I}(G_{krl}=s)\log\epsilon_{st}.\end{aligned} \tag{11}$$

Similarly, we can write

$$\begin{aligned}\log P(\mathbf{Z}|\Theta) &= \sum_{k=1}^{K}\mathrm{I}(Z_n=k)\log\pi_k\\ &+ \sum_{i\neq n}\sum_{k=1}^{K}\mathrm{I}(Z_i=k)\log\pi_k.\end{aligned} \tag{12}$$

Therefore, considering the second terms in (11) and (12) as constants and taking the expectation in (10), we obtain:

$$\begin{aligned}\log q^*(Z_n) &= \sum_{k=1}^{K}\mathrm{I}(Z_n=k)\Big\{\mathbb{E}_{q^*(\boldsymbol{\pi})}(\log\pi_k)\\ &+ \sum_{r=1}^{R}\sum_{l=1}^{L_r}\sum_{s\in\mathcal{S}}\sum_{t\in\mathcal{S}}\mathbb{E}_{q^*(G_{krl})}\big(\mathrm{I}(G_{krl}=s)\big)\mathrm{I}(X_{nrl}=t)\,\mathbb{E}_{q^*(\boldsymbol{\epsilon}_s)}(\log\epsilon_{st})\Big\} + C^{**}.\end{aligned}$$

So that $q^*(Z_n) \sim \text{Categorical}(\boldsymbol{\pi}_n^*)$ with parameters $\boldsymbol{\pi}_n^* = (\pi_{n1}^*,\ldots,\pi_{nK}^*)^T$ where $\sum_{k=1}^{K}\pi_{nk}^* = 1$ and

each π^*_{nk} is given by

$$\pi^*_{nk} = \frac{\exp\left\{ \mathbb{E}_{q^*(\boldsymbol{\pi})}(\log \pi_k) + \sum_{r=1}^{R}\sum_{l=1}^{L_r}\sum_{s\in\mathcal{S}}\sum_{t\in\mathcal{S}} \mathbb{E}_{q^*(G_{krl})}\big(\mathrm{I}(G_{krl}=s)\big)\mathrm{I}(X_{nrl}=t)\,\mathbb{E}_{q^*(\boldsymbol{\epsilon}_s)}(\log \epsilon_{st})\right\}}{\sum_{j=1}^{K}\exp\left\{ \mathbb{E}_{q^*(\boldsymbol{\pi})}(\log \pi_j) + \sum_{r=1}^{R}\sum_{l=1}^{L_r}\sum_{s\in\mathcal{S}}\sum_{t\in\mathcal{S}} \mathbb{E}_{q^*(G_{jrl})}\big(\mathrm{I}(G_{jrl}=s)\big)\mathrm{I}(X_{nrl}=t)\,\mathbb{E}_{q^*(\boldsymbol{\epsilon}_s)}(\log \epsilon_{st})\right\}}. \tag{13}$$

Using similar calculations as the ones above for $q^*(Z_n)$ and $q^*(\boldsymbol{\pi})$, we obtain the following posterior approximations for the remaining quantities of interest.

- $q^*(\boldsymbol{\mu}_{kr}) \sim \text{Dirichlet}(\boldsymbol{\beta}^*_{kr})$ where $\boldsymbol{\beta}^*_{kr}$ is the vector containing β^*_{krs} for every $s \in \mathcal{S}$ with

$$\beta^*_{krs} = \beta^0_s + \sum_{l=1}^{L_r} \mathbb{E}_{q^*(G_{krl})}\big(\mathrm{I}(G_{krl}=s)\big) \text{ for all } s\in\mathcal{S}. \tag{14}$$

- $q^*(G_{krl}) \sim \text{Categorical}(\boldsymbol{\mu}^*_{krl})$ where $\boldsymbol{\mu}^*_{krl} = \{\mu^*_{krls} : s \in \mathcal{S}\}$ with

$$\mu^*_{krls} = \frac{\exp\left\{ \sum_{n=1}^{N}\sum_{t\in\mathcal{S}} \mathbb{E}_{q^*(Z_n)}\big(\mathrm{I}(Z_n=k)\big)\mathrm{I}(X_{nrl}=t)\,\mathbb{E}_{q^*(\boldsymbol{\epsilon}_s)}(\log \epsilon_{st}) + \mathbb{E}_{q^*(\boldsymbol{\mu}_{kr})}(\log \mu_{krs})\right\}}{\sum_{v\in\mathcal{S}}\exp\left\{ \sum_{n=1}^{N}\sum_{t\in\mathcal{S}} \mathbb{E}_{q^*(Z_n)}\big(\mathrm{I}(Z_n=k)\big)\mathrm{I}(X_{nrl}=t)\,\mathbb{E}_{q^*(\boldsymbol{\epsilon}_v)}(\log \epsilon_{vt}) + \mathbb{E}_{q^*(\boldsymbol{\mu}_{kr})}(\log \mu_{krs})\right\}} \tag{15}$$

- $q^*(\boldsymbol{\epsilon}_s) \sim \text{Dirichlet}(\boldsymbol{\gamma}^*_s)$ where $\boldsymbol{\gamma}^*_s = \{\gamma^*_{st} : t \in \mathcal{S}\}$, where

$$\gamma^*_{st} = \gamma^0_{st} + \sum_{n=1}^{N}\sum_{r=1}^{R}\sum_{l=1}^{L_r}\sum_{k=1}^{K} \mathbb{E}_{q^*(Z_n)}\big(\mathrm{I}(Z_n=k)\big)\,\mathbb{E}_{q^*(G_{krl})}\big(\mathrm{I}(G_{krl}=s)\big)\mathrm{I}(X_{nrl}=t) \tag{16}$$

Step 4. Expectations and updates

Let ψ be the digamma function defined as

$$\psi(x) = \frac{d}{dx}\log\Gamma(x),$$

which can be easily calculated via numerical approximation. The values of the expectations in (8) and (13)-(16), taken with respect to the approximated distributions, are given as follows.

$$\mathbb{E}_{q^*(Z_n)}\big(\mathrm{I}(Z_n=k)\big) = \pi^*_{nk}$$

$$\mathbb{E}_{q^*(G_{krl})}\big(\mathrm{I}(G_{krl}=s)\big) = \mu^*_{krls}$$

$$\mathbb{E}_{q^*(\boldsymbol{\epsilon}_s)} = \psi(\gamma^*_{st}) - \psi\Big(\sum_{t\in\mathcal{S}} \gamma^*_{st}\Big)$$

$$\mathbb{E}_{q^*(\boldsymbol{\mu}_{kr})}(\log \mu_{krs}) = \psi(\beta^*_{krs}) - \psi\Big(\sum_{s\in\mathcal{S}} \beta^*_{krs}\Big)$$

$$\mathbb{E}_{q^*(\boldsymbol{\pi})}(\log \pi_k) = \psi(\alpha^*_k) - \psi\Big(\sum_{k=1}^{K} \alpha^*_k\Big)$$

Using the results above regarding the expectations, we update the parameters of the approximated distributions iteratively as follows.

We initialize $\boldsymbol{\gamma}^*_s$, $\boldsymbol{\beta}^*_{kr}$ and $\boldsymbol{\pi}^*_n$ with some arbitrary values (e.g., $\boldsymbol{\gamma}^{*(0)}_s = \boldsymbol{\gamma}^0_s$ and $\boldsymbol{\beta}^{*(0)}_{kr} = \boldsymbol{\beta}^0$). At each iteration c we compute:

1. $\boldsymbol{\alpha}^{*(c)}$ using $\boldsymbol{\pi}^{*(c-1)}_n$
2. $\boldsymbol{\mu}^{*(c)}_{krl}$ using $\boldsymbol{\gamma}^{*(c-1)}_s$, $\boldsymbol{\pi}^{*(c-1)}_n$ and $\boldsymbol{\beta}^{*(c-1)}_{kr}$
3. $\boldsymbol{\beta}^{*(c)}_{kr}$ using $\boldsymbol{\mu}^{*(c)}_{krl}$
4. $\boldsymbol{\gamma}^{*(c)}_s$ using $\boldsymbol{\mu}^{*(c)}_{krl}$ and $\boldsymbol{\pi}^{*(c-1)}_n$
5. $\boldsymbol{\pi}^{*(c)}_n$ using $\boldsymbol{\mu}^{*(c)}_{krl}$, $\boldsymbol{\gamma}^{*(c)}_s$ and $\boldsymbol{\alpha}^{*(c)}$

We then conduct many iterations of 1-5 until the convergence of the ELBO in (Eq. 2 of the main text).

1.2 Adjustment of imputed CpG states and naive imputation

In the cases when there are no observed values at a given CpG position for all cells in an estimated cluster, our probablistic model fills this value with the value that is the most common in the region of that CpG in that cluster. However, if this region is not a differentially methylated region, it may be better to just fill in with the CpG values from other clusters. Incorporating this mechanism in the Epiclomal model would require having another node influencing node G that depends on each CpG position, along with a trade-off mechanism that considers both the influence of other CpGs in the region for the cluster, and the influence of the observed values for the same CpG in other clusters. We reasoned that this would be an unnecessarily complicated and expensive addition to our model. Therefore, we added a post-processing "imputation adjustment" step in which we go through every region, and, if the prediction implies that this region is differentially methylated, we do not change anything; otherwise, for all the imputed CpGs that were completely missing in the cluster, we use the median value of all cells or 0 (unmethylated) if this median is 0.5 or undefined. We call a region

differentially methylated if there is a pair of clusters for which the G vectors for the region have Pearson correlation coefficient less than 0.5. Figure 3e and Figure 5 use this adjustment strategy for the EpiclomalRegion-adjusted case.

In order to assess Epiclomal's imputation of the missing CpG states, we also calculate a naive method of imputation. Using Epiclomal's predicted clusters, we infer a missing value with the median (or 0 if this is 0.5) of the observed CpG states in the same cluster, for the same locus. If there are no observed CpG states, then we use the median (or 0) of all cells. If there are still no observed states, we use 0.

1.3 Computational complexity

The Epiclomal methods are computationally more expensive than the non-probabilistic methods, but can provide some advantages in better prediction of the number of clusters (see, for example, Figure 6**e** and panel **c** of Supporting Figures B to I in S1 Figs), cell-to-cluster assignments (see V-measure in, for example, Figure 4) or epiclone frequency prediction (see Figures 3**c** and 6**d**). EpiclomalRegion is more computationally intensive than EpiclomalBasic, but because it imposes more structure into the data modelling it leads to overall better results (see, for example, Figures E**a**, E**c** and E**e** in S1 Figs).

1.4 Implementation

Epiclomal was implemented in Python 3. The remaining methods (data pre-processing, synthetic data generator, non-probabilistic methods and evaluation measures) were implemented in R 3.5. The computational framework consists of several pipelines run through the snakemake workflow. Our software is available at https://github.com/shahcompbio/Epiclomal.

2 Synthetic data generator

We generate synthetic data assuming the true cluster-specific methylation profiles differ only at certain regions following a phylogenetic tree structure.

For a given set of parameters as described in Table 2 of the main text, we generate a simulated data set of single-cell DNA methylation by doing as follows.

1. We start by generating the K vectors of true hidden methylation states according to the following steps.

   i. For a total number of loci M, we generate R regions with balanced sizes sampled from a multinomial distribution of size M and equal probabilities $1/R$;

ii. Each vector $\boldsymbol{\mu}_{1r}$ containing the probability of a given locus to be methylated in region r of cluster $k = 1$ is generated from a Dirichlet distribution.

iii. Each entry of the vector of hidden states (methylated or unmethylated) for cluster $k = 1$, $\mathbf{G}_1$, is generated by sampling from a Bernoulli distribution with probability of success and failure given by the $\boldsymbol{\mu}_{1r}$'s.

iv. We now generate the second vector of true methylation states, $\mathbf{G}_2$, by first setting $\mathbf{G}_2 = \mathbf{G}_1$ and then flipping the methylation states of a proportion of loci from a set of randomly picked regions, which size cannot exceed $R/K - 1$.

v. We generate $\mathbf{G}_k$ for $k = 3, \ldots, K$ by first randomly picking an ancestor vector of methylation states $\mathbf{G}_{k-1}$. We set $\mathbf{G}_k = \mathbf{G}_{k-1}$ and then flip the methylation states of a proportion of loci from a set of randomly picked regions, obtaining $\mathbf{G}_k$.

vi. If $\mathbf{G}_k$ is by chance equal to any previously generated vector we discard $\mathbf{G}_k$ and repeat Step v again.

2. We now generate the true vector of cell clustering assignments $\mathbf{Z}$ by sampling each entry from a multinomial distribution of size one and given cluster frequency probabilities.

3. We finally generate the observed data for each cell n as follows.

   i. If $Z_n = k$, the vector of observed methylation states for cell n is obtained by sampling the methylation state of each locus from a Bernoulli distribution with probabilities of success and failure depending on the true methylation state at that locus, G_{krl}, as in Equation (1).

   ii. To account for the presence of missing data we only keep a certain proportion of observations by choosing at random a locus and then keeping a random number (normally distributed with mean of 10 and standard deviation of 2) of observations to right and to the left of this site. We repeat this step many times till we obtain the desired proportion of observed data, which is one minus the desired missing proportion. By doing this procedure we are simulating sequencing reads that when aligned to the genome they cover multiple consecutive CpGs.

3 Evaluation measures

3.1 V-measure

V-measure is our main measure of clustering performance given a ground-truth clustering. V-measure assigns a score between 0 (random clustering) and 1 (perfect clustering) to a predicted set of cell-to-cluster assignments, when compared to the ground-truth clustering.

V-measure is calculated as the harmonic mean between homogeneity (h) and completeness (c): $V = \frac{2hc}{h+c}$. A predicted clustering has homogeneity 1 if all of the predicted clusters contain only data points which are members of a single true class. A predicted clustering result has completeness 1 if it assigns all of those datapoints that are members of a single true class to a single predicted cluster. Exact formulae of these measures are given in Rosenberg and Hirschberg [3]. We used the Python package scikit-learn to calculate V-measure.

3.2 Number of clusters

The number of predicted clusters is sometimes reported as an additional evaluation measure and compared with the ground-truth or expected number of clusters. This measure does not evaluate how correctly the cells are grouped together. Therefore, it is possible for a prediction to result in the correct number of clusters, but with V-measure close to 0. Nevertheless, the number of predicted clusters is a relevant measure to report in applications involving cancer dynamics as it is of great importance to know the approximate number of tumour clones.

3.3 Cluster frequency mean absolute error

To calculate the cluster frequency MAE (mean absolute error), we assign to each cell n its corresponding true and predicted cluster frequency values p_n and $\hat{p}_n$, respectively, and compute $1/N \sum_{n=1}^{N} |p_n - \hat{p}_n|$, where N is the total number of cells.

The true cluster frequency for a given cell n can be given (in the case of the synthetic data simulations) or calculated as $p_n = \sum_{i=1}^{N} I(c_i = c_n)/N$, where I is the indicator variable that returns true (or 1) if the true cluster of cell i c_i is the same as the true cluster of cell n c_n. In other words, we calculate the proportion of cells that have the same true clustering assignment as cell n (including n).

The predicted cluster frequency value $\hat{p}_n$ for a given cell n is calculated by averaging over N the estimated clustering assignment parameters π^*_{nk}, see Section 1.1, $\hat{p}_n = \sum_{n=1}^{N} \pi^*_{nk}/N$. This is very similar to calculating the proportion of cells within the same predicted cluster, but since the predicted clustering assignments are probabilities, the result will be slightly different.

3.4 Hamming distance

For each cell in a synthetic dataset, we calculate the hamming distance as the proportion of discordant entries between true and estimated vectors of methylation states, which correspond to the true and predicted clusters for that cell. This is done for all CpG loci, including observed and inferred loci. We then report the mean hamming distance over all cells.

3.5 Uncertainty true positive rate

To compute the uncertainty true positive rate (TPR) for Epiclomal predictions, we proceed as follows. First, we look at the Epiclomal's predicted vector of epigenotypes (matrix **G** in Figure 1 of the main text). For each epigenotype, we go through all the regions and build a list of regions that differ between at least two epiclones. Then, we build a list of "uncertain cells", that is, a list of cells that could belong to more than one cluster because of one or more of the different regions is completely missing. For each of the "uncertain cells" we also build a set of "candidate clusters", that is, all the clusters that cell could belong to. All the cells in the "uncertain cells" list should have a posterior clustering assignment probability of less than 1, roughly 1 divided by the number of possible clusters this cell could belong to (if a cell could belong to one of 2 clusters the probability should be roughly 0.5; if it could belong to one of 3 clusters the probability should be roughly 0.33). If this is the case, then the uncertainty with respect to this cell was predicted well and it is considered a true positive. The uncertainty TPR is calculated as the number of true positives divided by the number of uncertain cells. For example, if all the uncertain cells were predicted with the approximate correct posterior probability, the uncertainty TPR is 1. If all the uncertain cells were given a probability of 1, than the uncertainty TPR is 0. Finally, if the number of uncertain cells is 0, then the uncertainty TPR is undefined. This is an ad-hoc way to estimate the uncertainty TPRs and may not be easily extended to more complicated or noisy cases such as for real data, but it gives us a way to evaluate uncertainty for the simpler and more controlled scenarios considered in our simulations.

4 Supplementary Tables

Dataset	Overall PM	PM - CpGs ≥ 2	CpGs with ≥ 2	CpGs with data	Total CpGs
Smallwood2014 [4]	1.32 (0.74)	2.23 (0.9)	58.34 (5.89)	42.77 (8.99)	1,077,351
Hou2016 [5]	0.23 (0.12)	0.23 (0.12)	100 (0)	2.86 (1.67)	2,089,538
Farlik2016 [6]	0.08 (0.05)	5.13 (3.26)	1.45 (0.84)	1.1 (0.73)	2,291,383
Inhouse	0.76 (0.51)	1.44 (2.4)	41.63 (30.6)	20.62 (13.74)	2,079,646

Table A: **Additional information on the real datasets used in this work.** Column descriptions (in order of appearance) are as follows: (1) dataset names corresponding to three published datasets and the new in-house dataset; (2) median percentage and between parentheses the interquartile range (IQR) across cells of CpGs with partial methylation (PM) over all CpGs with data; (3) median percentage and IQR across cells of CpGs with PM over sites with at least two reads aligned to them; (4) median percentage and IQR across cells of CpGs with at least 2 reads aligned to them; (5) median percentage and IQR across cells of CpGs with observed data; (6) Total number of CpGs per cell in the functional regions considered for each data set before pre-processing.

Patient tumour ID	Tumour type	Plate ID	Passage	Number of cells
SA501	TNBC	px0582	X10A	45
SA501	TNBC	px0680	X10A	61
SA501	TNBC	px0443	X7A	48
SA501	TNBC	px0472	X7A	50
SA501	TNBC	px0837	X2	40
SA532	ER+PR-Her2+	px0544	X6	68
SA532	ER+PR-Her2+	px0650	X6	71
SA609	TNBC	px0738	X6	48
SA609	TNBC	px0739	X6	52
SA609	TNBC	px0740	X6	60
SA609	TNBC	px0741	X6	55

Table B: *Breast cancer xenograft sc-WGBS data from three patients (SA501, SA535 and SA609).* The InHouse data presented in main text Figure 7 correspond to cells from all plates except plate px0837 (xenograft passage 2), summing 558 cells in total. The data presented in the main text Figure 8 correspond to all SA501 plates, totalizing 244 cells.

References

[1] Michael I Jordan, Zoubin Ghahramani, Tommi S Jaakkola, and Lawrence K Saul. An introduction to variational methods for graphical models. *Machine learning*, 37(2):183–233, 1999.

[2] David M Blei, Alp Kucukelbir, and Jon D McAuliffe. Variational inference: A review for statisticians. *Journal of the American Statistical Association*, 112(518):859–877, 2017.

[3] Andrew Rosenberg and Julia Hirschberg. V-measure: A conditional entropy-based external cluster evaluation measure. In *Proceedings of the 2007 joint conference on empirical methods in natural language processing and computational natural language learning (EMNLP-CoNLL)*, 2007.

[4] Sébastien A Smallwood, Heather J Lee, Christof Angermueller, Felix Krueger, Heba Saadeh, Julian Peat, Simon R Andrews, Oliver Stegle, Wolf Reik, and Gavin Kelsey. Single-cell genome-wide bisulfite sequencing for assessing epigenetic heterogeneity. *Nature methods*, 11(8):817–820, 2014.

[5] Yu Hou, Huahu Guo, Chen Cao, Xianlong Li, Boqiang Hu, Ping Zhu, Xinglong Wu, Lu Wen, Fuchou Tang, Yanyi Huang, et al. Single-cell triple omics sequencing reveals genetic, epigenetic, and transcriptomic heterogeneity in hepatocellular carcinomas. *Cell research*, 26(3):304, 2016.

[6] Matthias Farlik, Florian Halbritter, Fabian Müller, Fizzah A Choudry, Peter Ebert, Johanna Klughammer, Samantha Farrow, Antonella Santoro, Valerio Ciaurro, Anthony Mathur, et al. Dna methylation dynamics of human hematopoietic stem cell differentiation. *Cell stem cell*, 19(6):808–822, 2016.
