# Supplementary material for: Epiclomal: Probabilistic clustering of sparse single-cell DNA methylation data
Source: PLoS Comput Biol. 2020 Sep 23;16(9):e1008270. doi: 10.1371/journal.pcbi.1008270 (PMC7546467; doi:10.1371/journal.pcbi.1008270)
Supplement: S1 Figs — (PDF) [file pcbi.1008270.s002.pdf]

Epiclomal: probabilistic clustering of sparse single-cell DNA methylation data

List of Supporting Figures

A Example of a DIC elbow plot 2
B Simulation results when varying the missing proportion 3
C Simulation results when varying the number of regions 4
D Simulation results when varying the number of cells 5
E Simulation results when varying the cell-to-cell variability 6
F Simulation results when varying the number of epiclones 7
G Simulation results when varying the cluster frequencies 8
H Simulation results when varying the number of loci 9
I Simulation results when varying the number of different regions . . . 10
J EpiclomalRegion posterior mean methylation probabilities 11
K EpiclomalRegion clustering on the Smallwood2014 data set 12
L EpiclomalRegion clustering on the Hou2016 data set 13
M EpiclomalRegion clustering on the Farlik2016 data set 14
N Average methylation heatmap for SA501 15
O Distributions of the selected regions for SA501 16
P EpiclomalRegion robustness to the Variational Bayes iterations. . . . 17
Q Subsampling results for Smallwood2014. 18

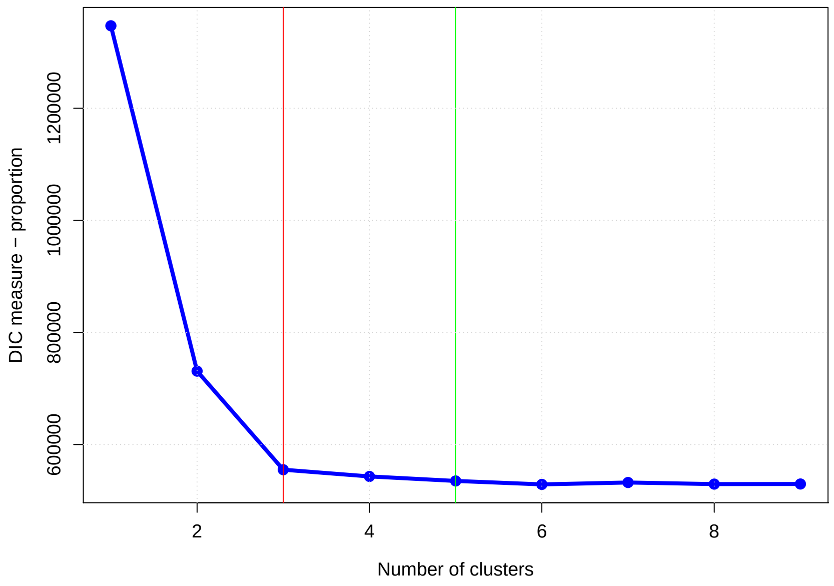

Figure A Example of a DIC elbow plot for EpiclomalRegion for the InHouse data set with 10 000 loci. Our DIC algorithm selects only number of clusters for which there is a decrease of at least 2% in DIC values, green vertical line. Then, the elbow value is picked, red vertical line.

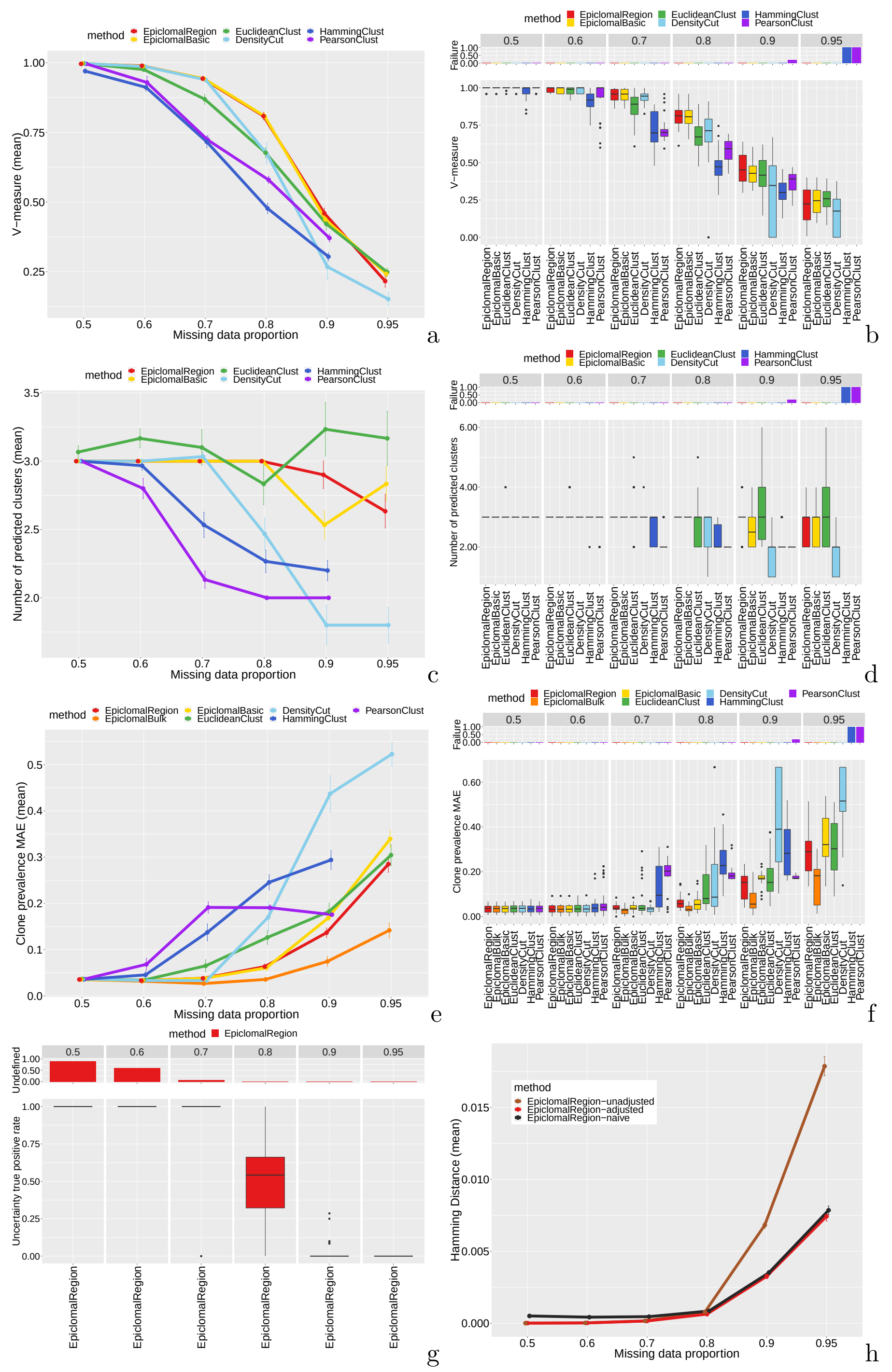

Figure B Simulation results when varying the missing proportion. Panels a and b show results for V-measure in two different ways: means and error bars in panel a, and boxplots in panel b. The barplots above the boxplots show the proportion of data sets for which a method failed to produce a result. Equivalently, panels c and d show results for the number of predicted clusters (epiclones). The correct number of clusters is 3. Panels e and f show results for the epiclone frequency (prevalence) MAE. Panel g shows boxplots for the uncertainty true positive rate (TPR), with the top panel showing the proportion of data sets for which the uncertainty TPR is undefined because there is no true uncertainty, see also Section 3.5 in S1 Material. Panel h shows the average hamming distance for three variants of EpiclomalRegion inferred methylation states: unadjusted, adjusted and naive, see Sections 1.2 and 3.4 in S1 Material.

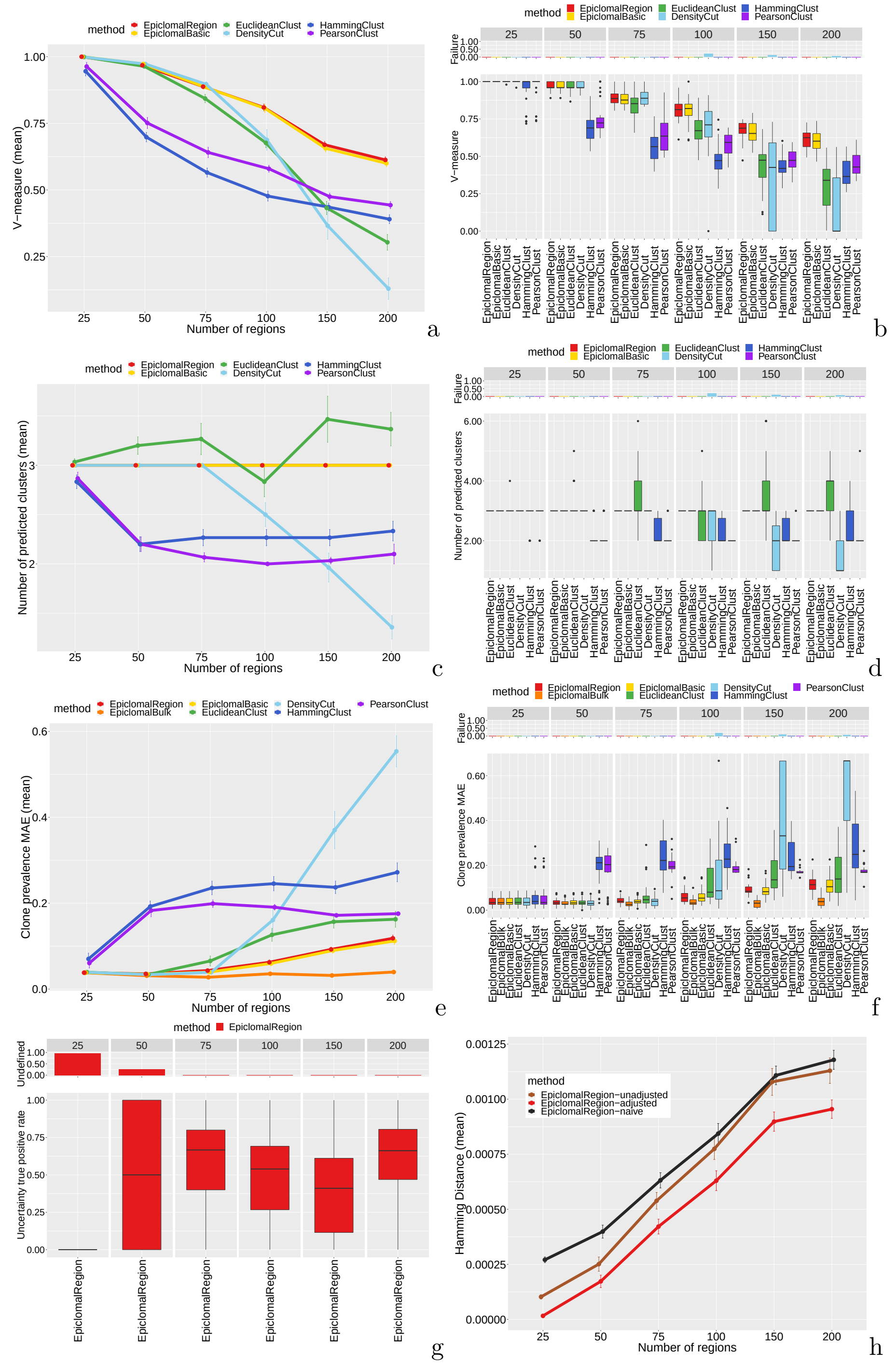

Figure C Simulation results when varying the number of regions. Plots as in Figure B. The larger the number of regions the smaller the differences among epiclones as the number of loci is fixed and data are generated with only one region changing states at each new cluster generation. The Epiclomal methods perform better than the other methods and correctly predict the number of clusters (correct number is 3, panel c). As expected, all methods perform worse for the largest number of regions because there is less difference between epiclones (200 regions correspond to 0.5% of the loci being different, while 25 regions correspond to 4% of the loci being different).

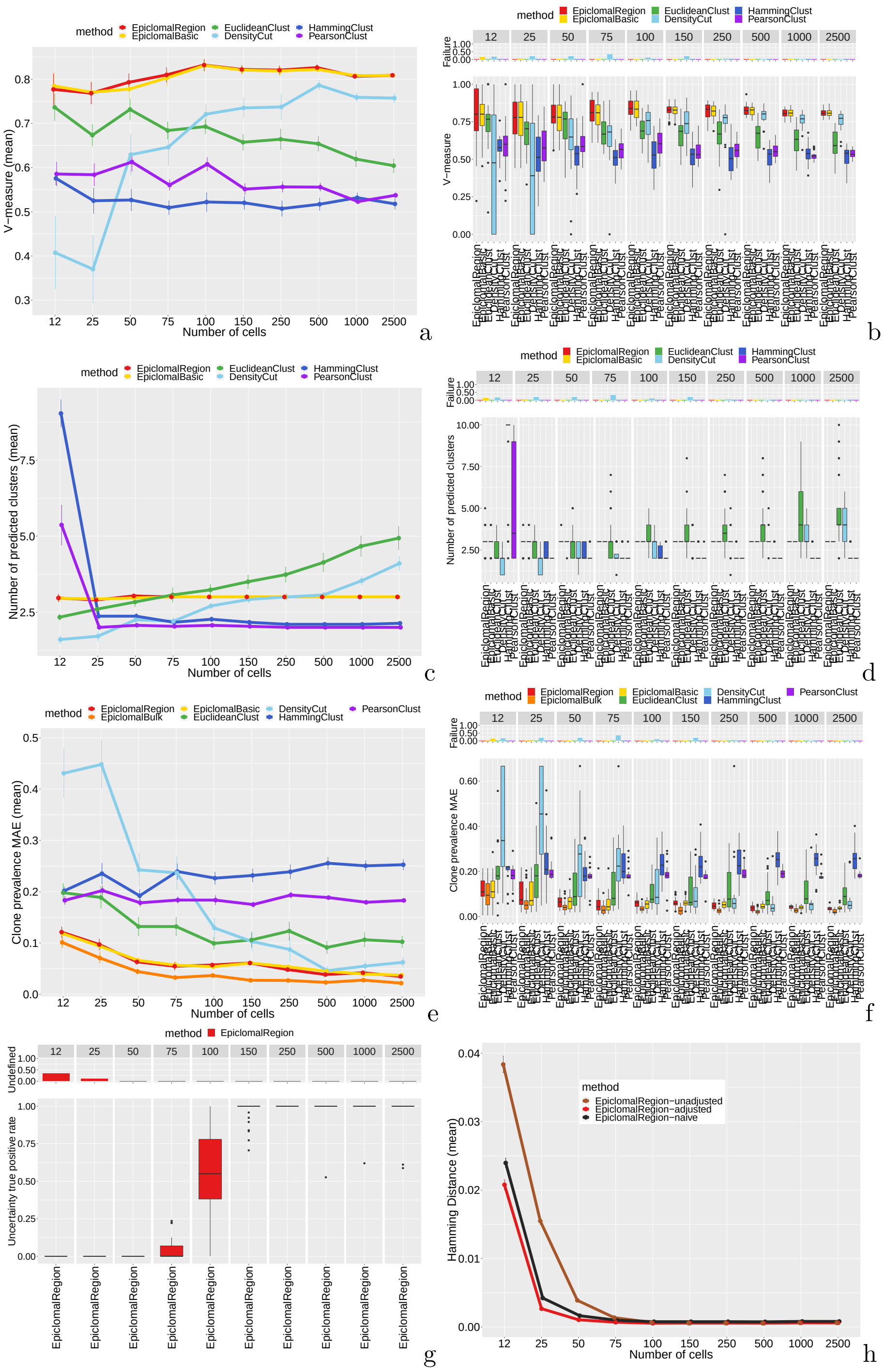

Figure D Simulation results when varying the number of cells. Plots as in Figure B. Increasing the number of cells does not improve the overall V-measure, except for DensityCut, but it reduces the variability of V-measure values. Epiclomal methods produce better V-measures in this case than the other methods. Panel e) shows that EpiclomalBulk produced the best estimates of epiclone frequencies (prevalences). Starting at about 150 cells EpiclomalRegion was able to obtain an uncertainty true positive rate close to one (panel g). The correct number of clusters is 3.

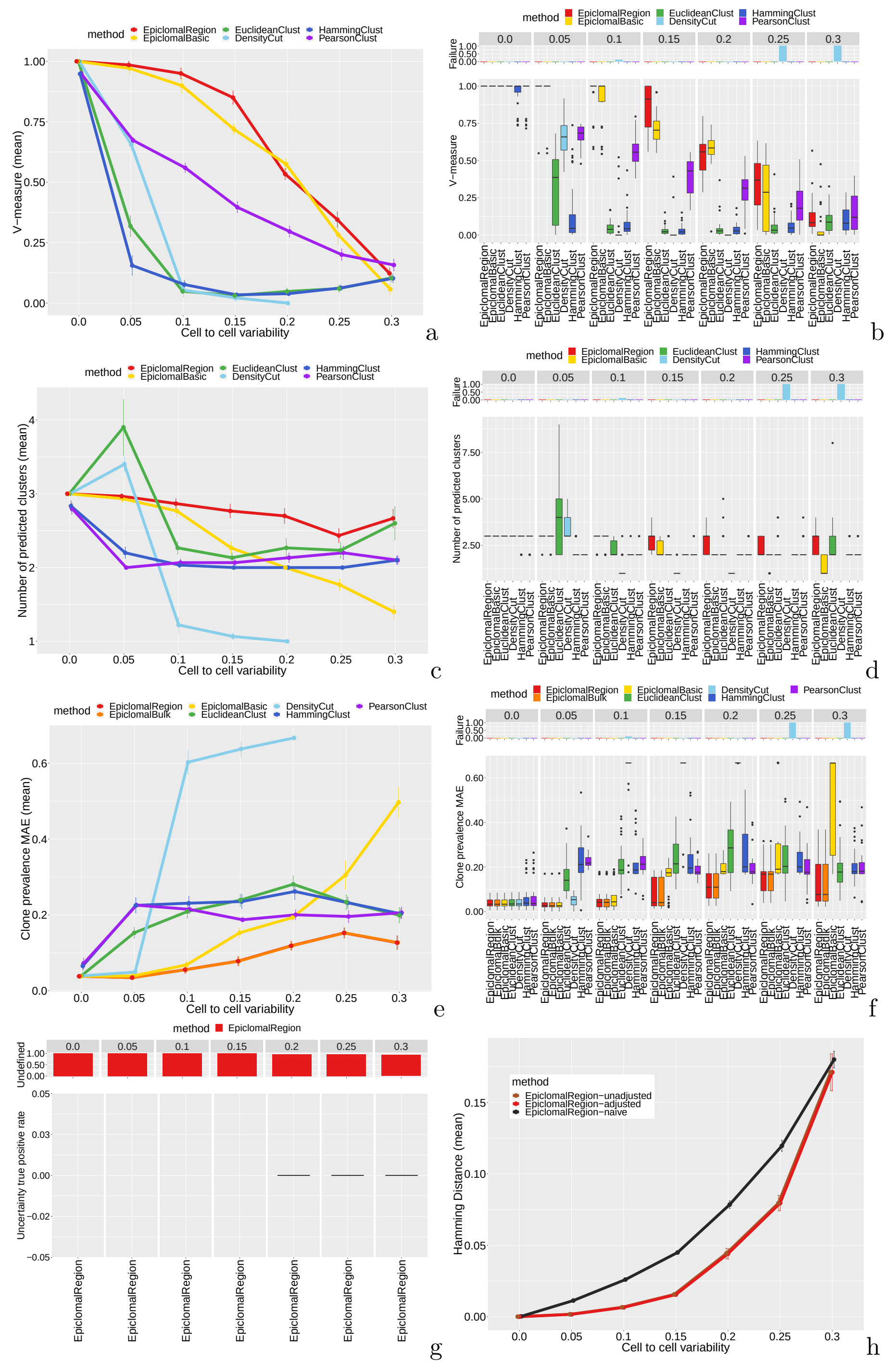

Figure E Simulation results when varying the cell-to-cell variability. Plots as in Figure B. The Epiclomal methods perform significantly better than the other methods when the cell-to-cell variability is between 0.05 to 0.20. A cell-to-cell variability of 0.20 means that at each CpG location that is not in the separating regions (regions that are different among clusters), 20% of random cells are in the opposite methylation state than the remaining 80%. Hence a variability of 0.50 means that all the non-separating CpGs have completely random methylation states. When the variability is large (≥ 0.25), all methods perform poorly. The correct number of clusters is 3.

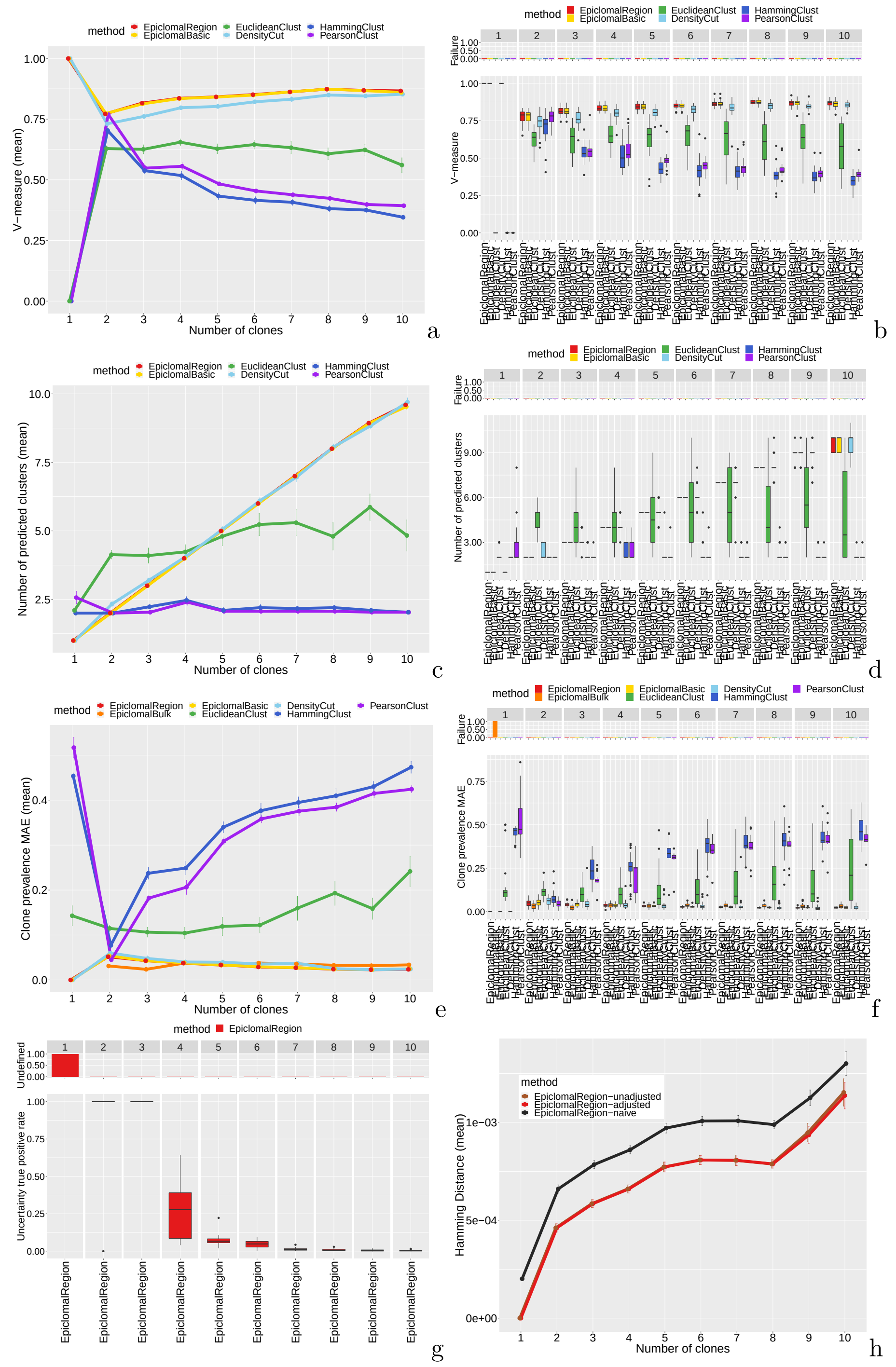

Figure F Simulation results when varying the number of epiclones (clusters) from 1 to 10. Plots as in Figure B. Epiclomal methods perform slightly better than DensityCut and much better than the other methods in terms of V-measure. Epiclomal and DensityCut are the only methods that can correctly predict the number of clusters (panel c).

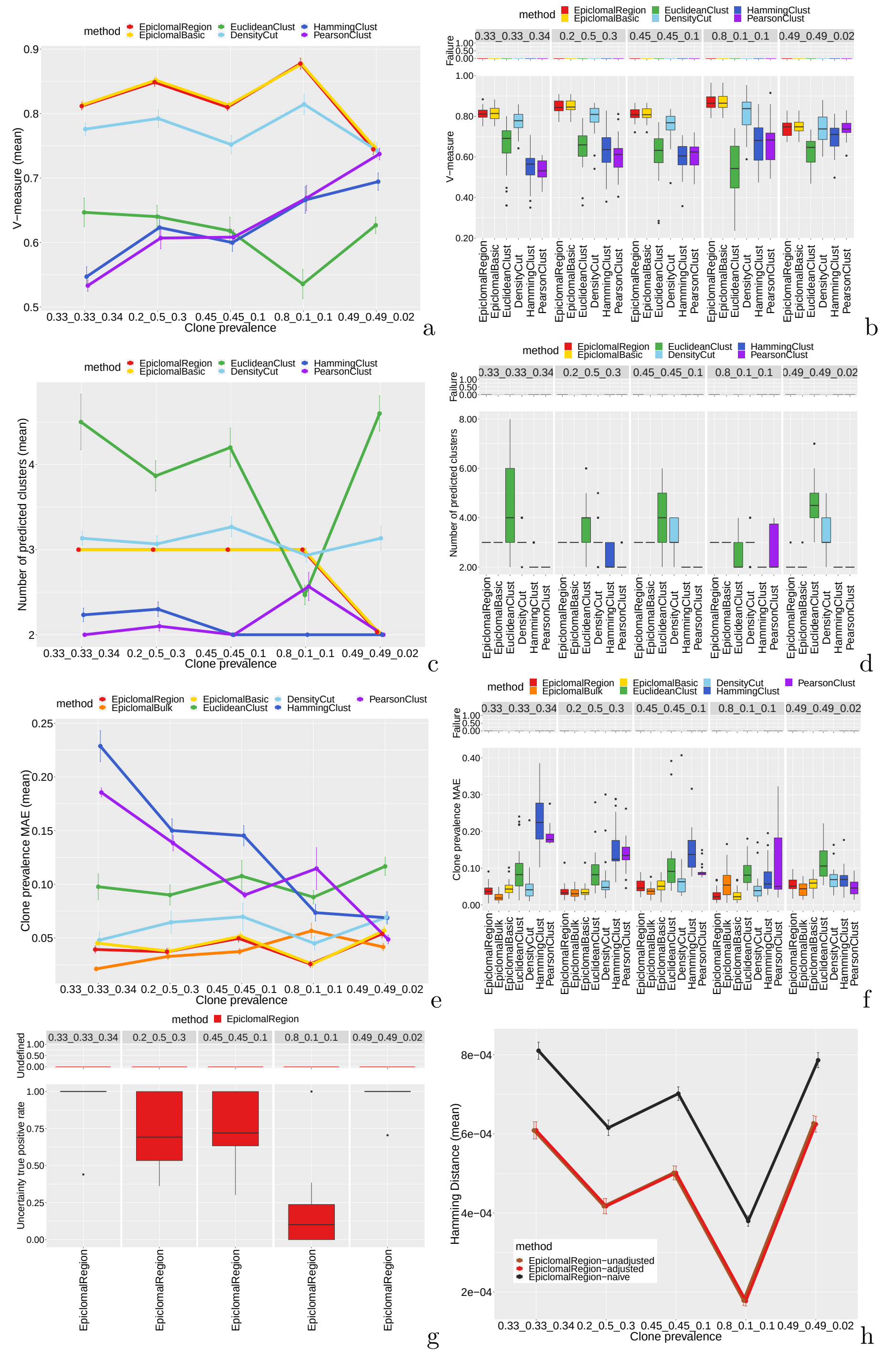

Figure G Simulation results when varying the cluster frequencies (prevalences) from equal to very imbalanced. The number of clusters is 3. Plots as in Figure B. EpiclomalRegion and EpiclomalBasic give better V-measures than the other methods, and they correctly predict three clusters, except in the case where one of the clusters has only 2% of the cells. Interestingly, DensityCut does predict three clusters for this difficult case. The uncertainty true positive rate for EpiclomalRegion is above 0.75 for all cases except the case where two of the clusters have only 10% of the cells each.

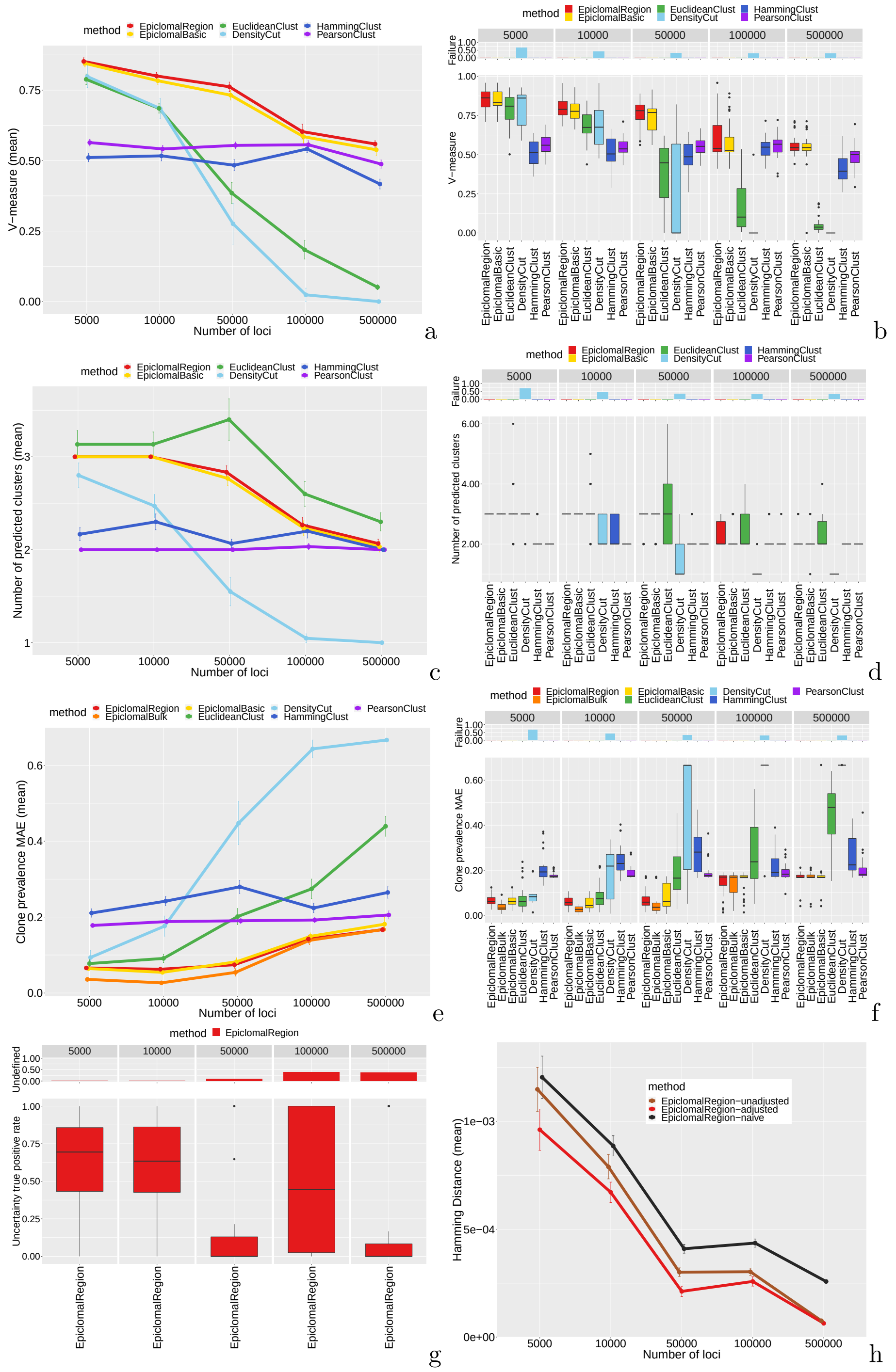

Figure H Simulation results when varying the number of loci. Plots as in Figure B. As we increase the number of loci the number of regions also increase, but their sizes remain fixed and so the amount of CpGs that make the clusters different. The performance of HammingClust and PearsonClust remains somewhat constant as we increase the number of loci, while the other methods show a decreasing pattern in performance. However, the Epiclomal methods still perform better in all cases than all the other methods, especially for 5 000, 10 000 and 50 000 loci. Therefore, this provides support to the strategy of selecting a smaller number of loci (under 50 000) in order to keep the true signal and eliminate noise when analyzing a real data set. The correct number of clusters is 3.

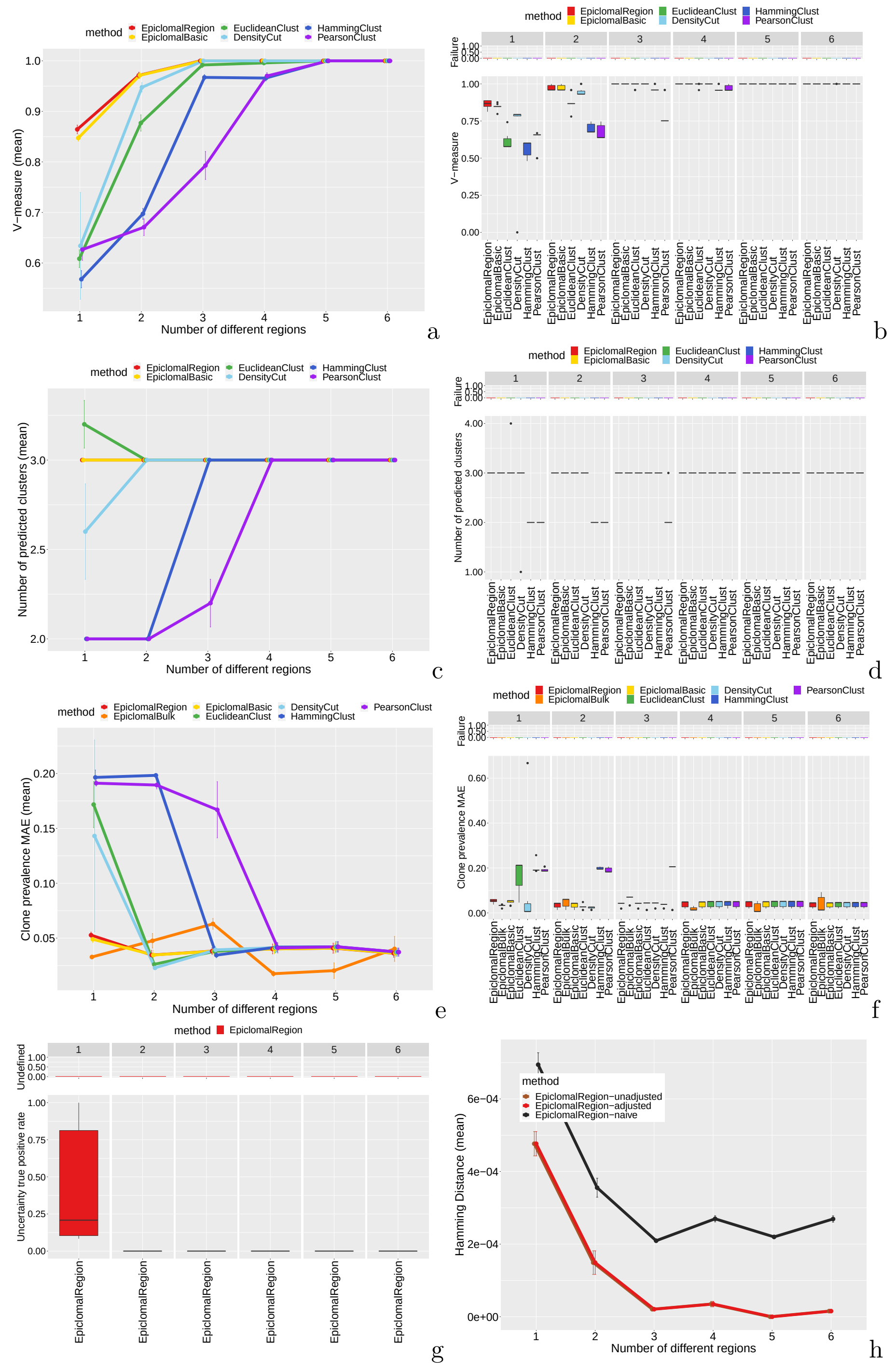

Figure I Simulation results when varying the number of regions that are different between clusters. Plots as in Figure B. As we increase the number of regions, the problem becomes easier for all methods, as there are more different loci. Epiclomal outperforms the other methods when there are fewer different regions between clusters.

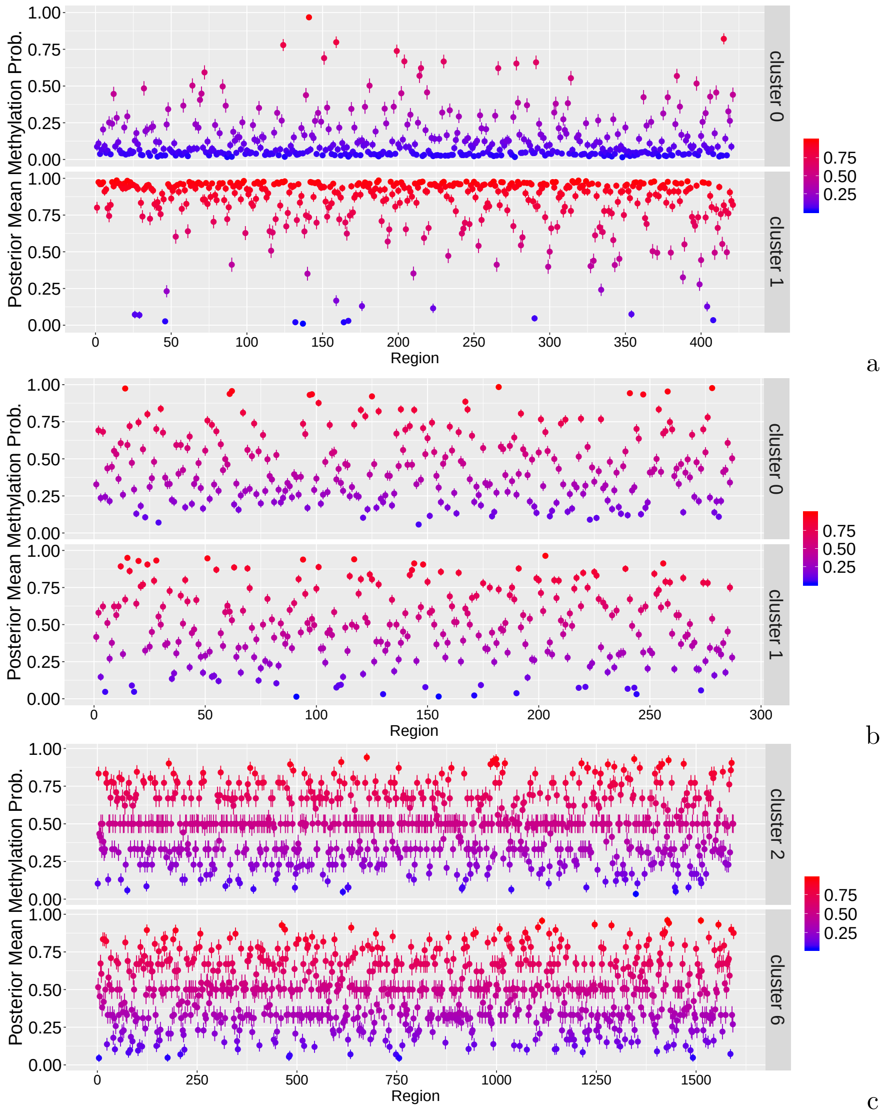

Figure J EpiclomalRegion posterior mean methylation probabilities across regions for the inferred clusters in (a) Smallwood2014, (b) Hou2016 and (c) Farlik2016 datasets filtered to include most variable regions and obtain $\sim 10\,000$ loci. For the Farlik2016 dataset we show the results for 2 clusters out of the 6 inferred by EpiclomalRegion as the results for remaining ones are similar. The dots corresponds to the posterior mean probabilities of a CpG being methylated (i.e., the posterior mean of μ_{kr}, see Figure 1b in the main text and Equation 14 in S1 Material) and the bars to one posterior standard deviation below and above the mean.

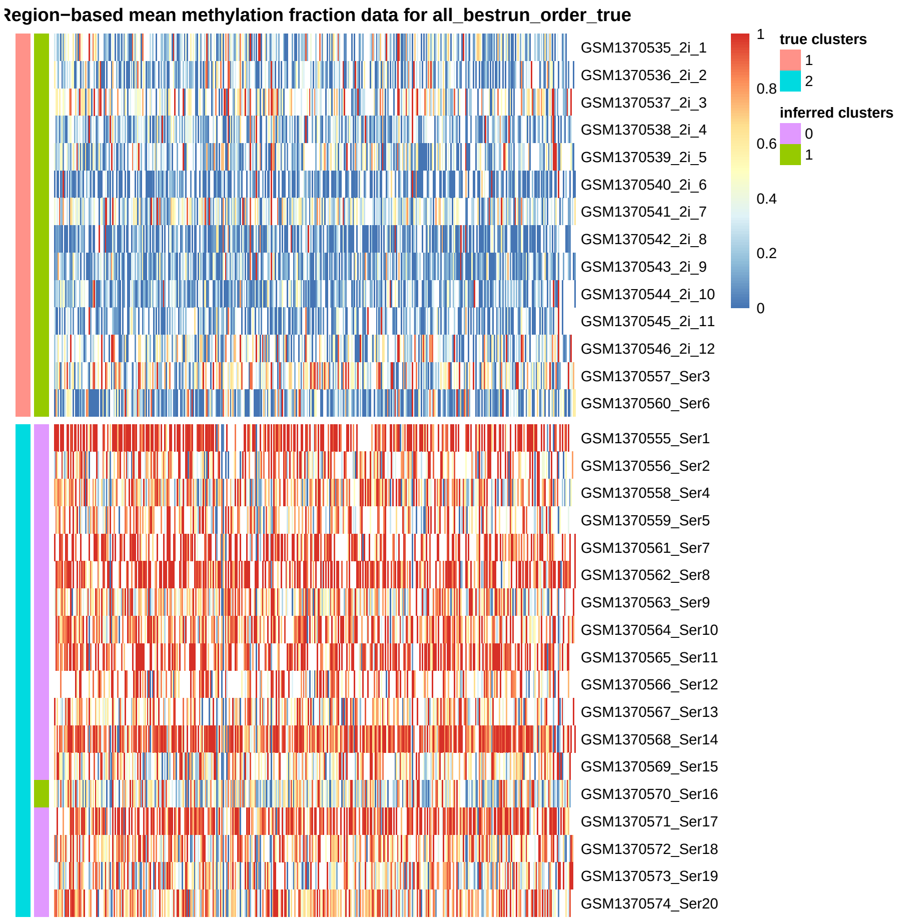

Figure K EpiclomalRegion clustering on the Smallwood2014 data set, filtered to include the most variable regions and obtain $\approx 10\,000$ loci (421 regions, average missing proportion 0.69, 32 cells). EpiclomalRegion returned the correct number of clusters (2), but missclassified one cell (GSM1370570_Ser16), V-measure = 0.83. The configuration with the misclassified cell obtained a slightly better DIC score than the ground truth.

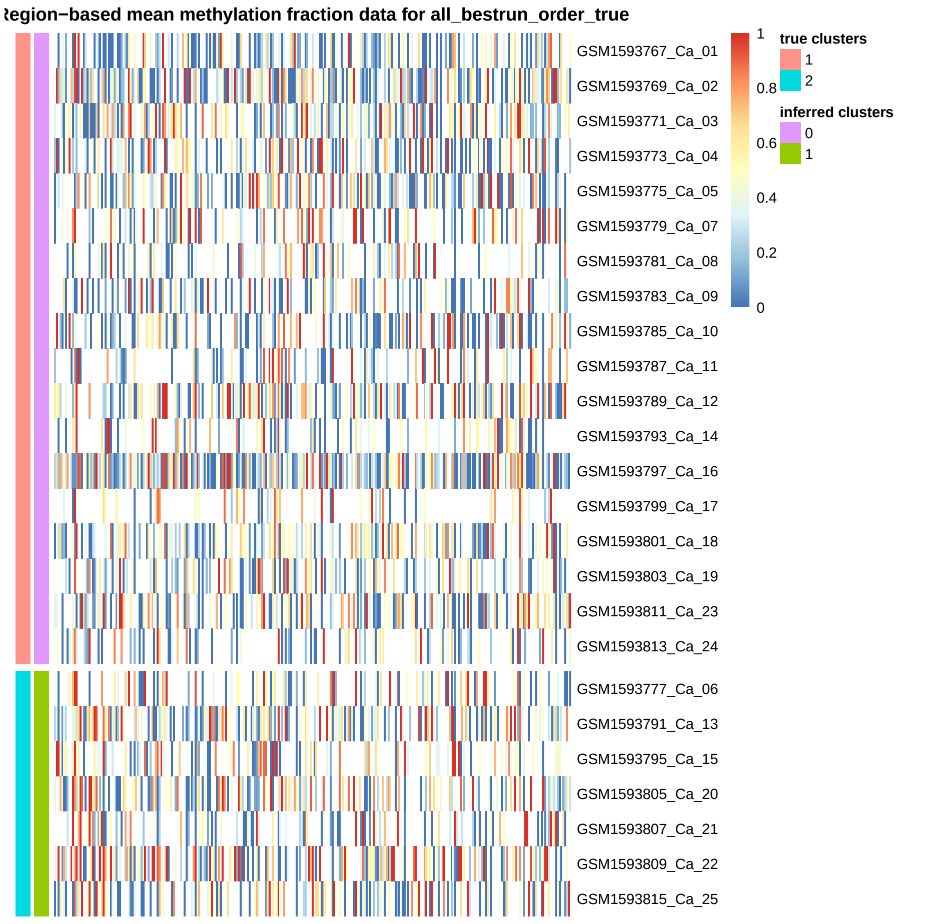

Figure L EpiclomalRegion clustering on the Hou2016 data set, filtered to include the most variable regions and obtain $\approx 10\,000$ loci (287 regions, average missing proportion 0.87, 25 cells). EpiclomalRegion correctly classified all cells, V-measure $= 1$.

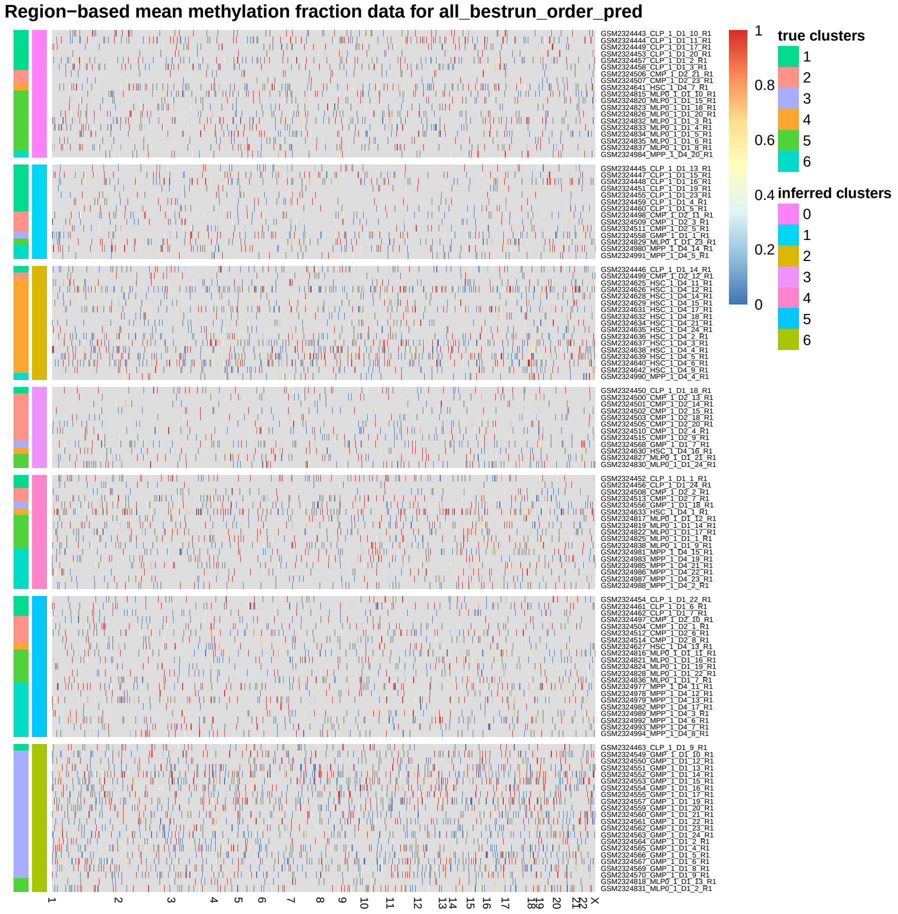

Figure M EpiclomalRegion clustering on the Farlik2016 data set, filtered to include the most variable regions and obtain ≈ 10 000 loci. For this set, we eliminated the regions that had on average more than 98% missing CpGs. EpiclomalRegion obtained 7 clusters (ground-truth is 6) and V-measure 0.36.

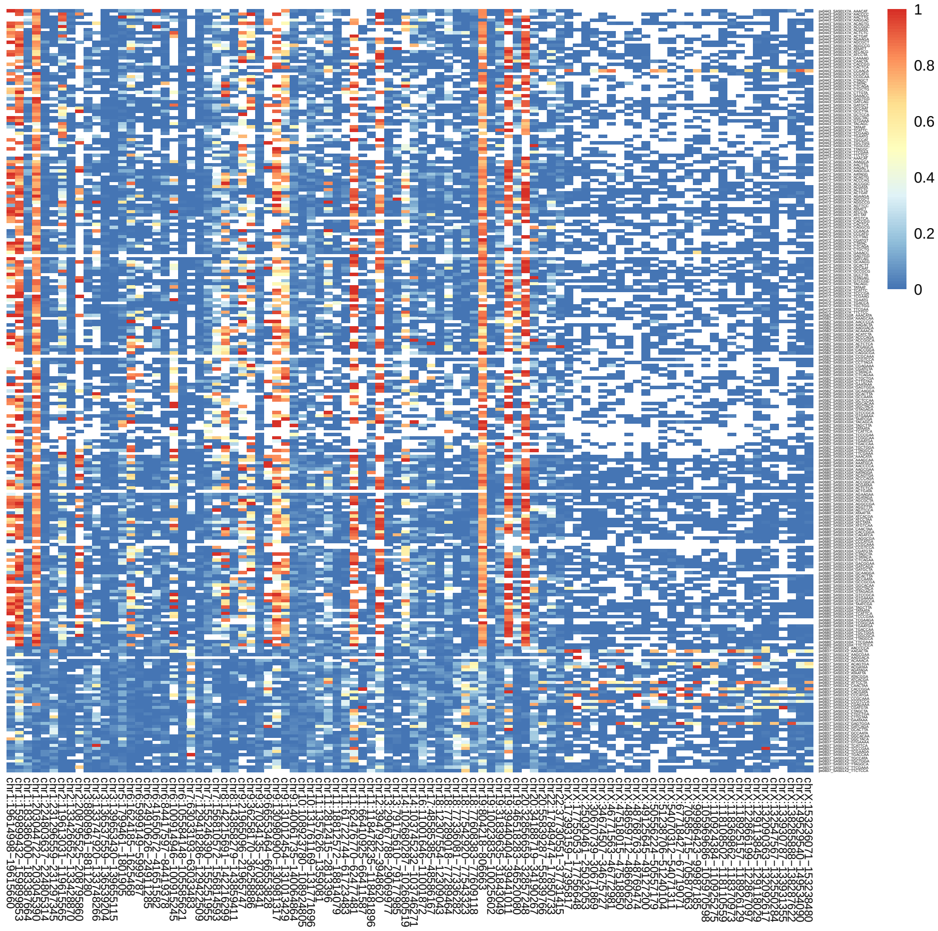

Figure N Average methylation for the all the 244 SA501 cells and the 94 NMF-selected regions (CGIs), unclustered. The x-axis shows the region chromosome, start and end.

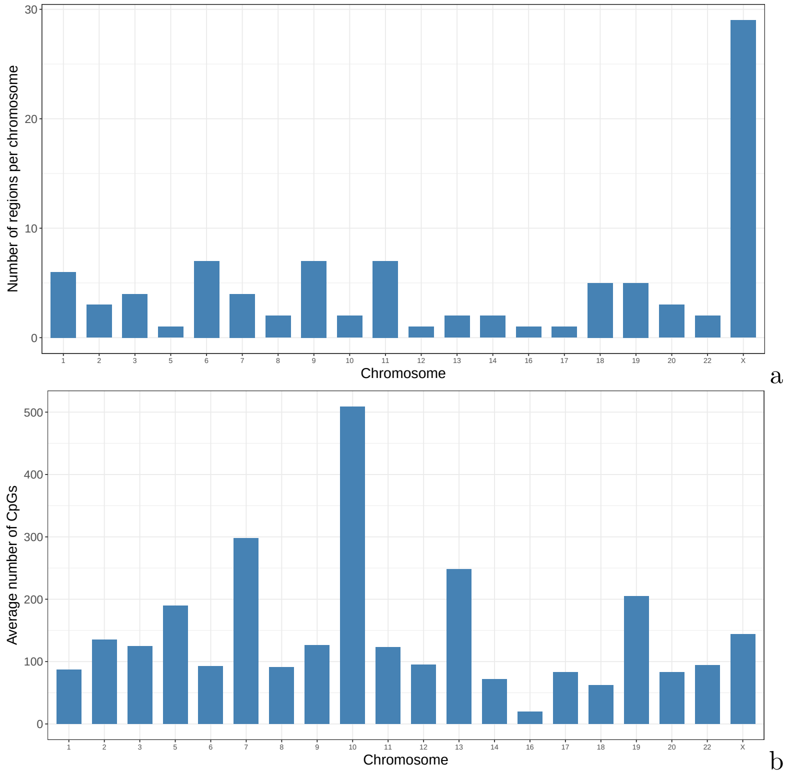

Figure O a) Distribution of the 94 NMF-selected regions (CGIs) for SA501 data across the genome. b) Average number of CpGs across regions per chromosome.

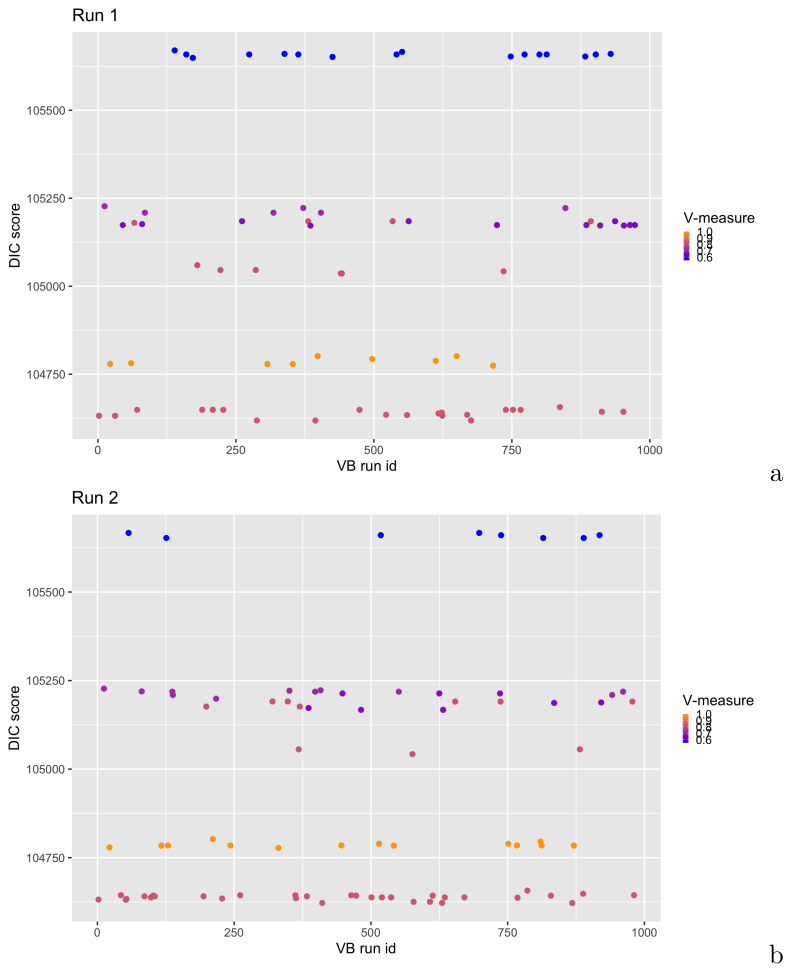

Figure P a) and b) correspond to two independent EpiclomalRegion runs across different initializations on the Smallwood2014 data set (filtered to include the most variable regions and obtain $\approx 10\,000$ loci). For each Variational Bayes initialization that resulted in two clusters, we plot the DIC score against the initialization run ID. The resulting V-measure is depicted in the corresponding colour. Both runs show a similar pattern of DIC score versus run ID and resulted in the same optimal clustering.

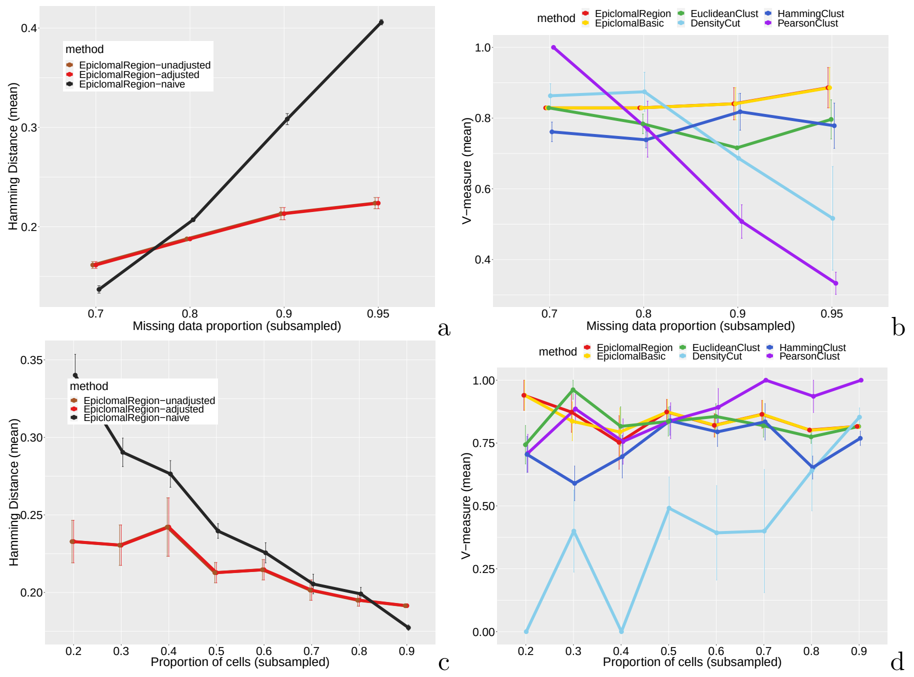

Figure Q Subsampling the Smallwood2014 data set by missing probability (panels a and b); 0.7 means that we removed enough CpGs to obtain this missing proportion), and by number of cells (panels c and d); 0.2 keeps only 20% of the initial cells). For each subsampling step, 10 random datasets were generated.
